# From betaines to anionic N-heterocyclic carbenes. Borane, gold, rhodium, and nickel complexes starting from an imidazoliumphenolate and its carbene tautomer

**DOI:** 10.3762/bjoc.12.264

**Published:** 2016-12-08

**Authors:** Ming Liu, Jan C Namyslo, Martin Nieger, Mika Polamo, Andreas Schmidt

**Affiliations:** 1 Clausthal University of Technology, Institute of Organic Chemistry, Leibnizstrasse 6, D-38678 Clausthal-Zellerfeld, Germany; 2 University of Helsinki, Department of Chemistry, P.O. Box 55, FIN-00014 Helsinki, Finland

**Keywords:** anionic ligand, carbene tautomer, imidazol-2-ylidene, mesoionic compound, mesomeric betaine

## Abstract

The mesomeric betaine imidazolium-1-ylphenolate forms a borane adduct with tris(pentafluorophenyl)borane by coordination with the phenolate oxygen, whereas its NHC tautomer 1-(2-phenol)imidazol-2-ylidene reacts with (triphenylphosphine)gold(I) chloride to give the cationic NHC complex [Au(NHC)_2_][Cl] by coordination with the carbene carbon atom. The anionic N-heterocyclic carbene 1-(2-phenolate)imidazol-2-ylidene gives the complexes [K][Au(NHC^−^)_2_], [Rh(NHC^−^)_3_] and [Ni(NHC^−^)_2_], respectively. Results of four single crystal analyses are presented.

## Introduction

**Figure 1:**
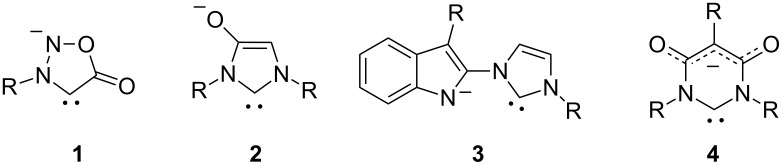
Examples of anionic N-heterocyclic carbenes.

Since the first isolation of a stable N-heterocyclic carbene (NHC) [1] in 1991 this compound class has provided numerous highly efficient ligands of NHC-metal catalysts for cross-coupling reactions [2–7], versatile organocatalysts [8–10], and starting materials for heterocycle syntheses [11–13]. Consequently books [14–16] and reviews cover the range from NHC structures in the light of their early history [17], syntheses [6,18], coordination chemistry [19–20], and catalysis [21–22], to biological activities of NHC complexes [23–24]. In the last decade, attention has also been directed to anionic N-heterocyclic carbenes [25]. In this context, mesomeric betaines are interesting from two viewpoints. They are not only able to undergo tautomerisations to neutral NHCs and thus provide a safe storage form of otherwise unstable species [26–27], but proved also to be suitable starting materials for the generation of anionic NHCs by deprotonation. As examples of the latter mentioned species, sydnone anions **1** [28], imidazole-2-ylidene-4-olate **2** [29] as well as its 4-aminide derivatives [30] have been prepared from mesoionic compounds (Figure 1). The carbenes **3** [31] and **4** [32–33] originate from a conjugated ylide and a cross-conjugated mesomeric betaine, respectively. A review elucidates the interconversions of mesomeric betaines to different types of N-heterocyclic carbenes (NHC, aNHC, rNHC, MICs) and vice versa [34].

Herein, conjugated mesomeric betaine 2-(1-butyl-1*H*-imidazolium-3-yl)phenolate, its tautomeric NHC and its anionic NHC are shown to serve as monodentate and bidentate ligands, respectively.

## Results and Discussion

**Scheme 1:**
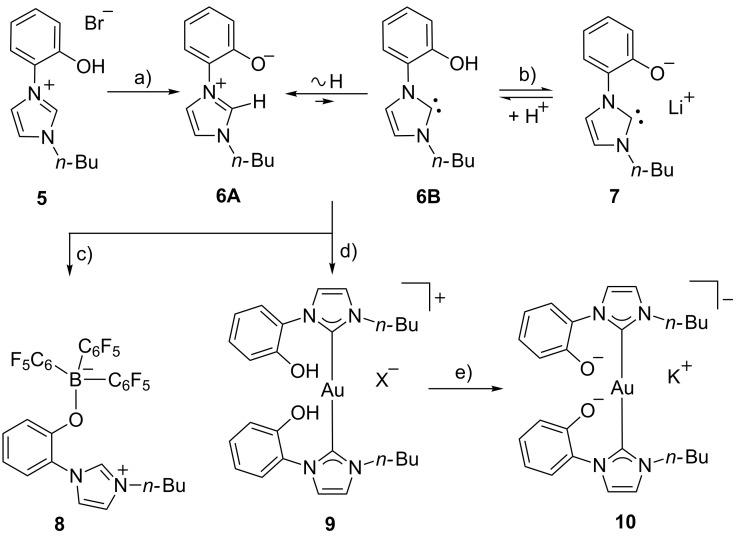
A postulated mesomeric betaine – NHC equilibrium (**6A**/**6B**) and formation of an anionic NHC **7**. Formation of borane adduct **8** of tautomer **6A**, a cationic gold complex **9** of tautomer **6B**, and an anionic gold complex **10** of anionic NHC **7**. a) K_2_CO_3_, MeOH, reflux, 4 h, 98%. b) LiN(TMS)_2_, pyridine, rt, 100%. c) B(C_6_F_5_)_3_, dioxane, reflux, 43%. d) (PPh_3_)AuCl, THF, reflux, 60%; X = Cl/Br. e) K_2_CO_3_, MeOH, 100%.

Salt **5** is available via a three-step procedure [35]. Deprotonation with potassium carbonate resulted in the formation of the tautomeric equilibrium of the mesomeric betaine **6A** and its NHC **6B** (Scheme 1). The NMR spectra measured in a variety of solvents (DMSO-*d*_6_, MeCN-*d*_3_, MeOD-*d*_4_, D_2_O, THF-*d*_8_) show that the polar mesomeric betaine **6A** is the only detectable tautomer under these conditions; unfortunately the addition of less polar solvents induces a precipitation from solution so that detection of the NHC tautomer by NMR spectroscopy is not possible under these conditions. Base screening revealed that the anionic N-heterocyclic carbene **7** can be generated in quantitative yield at rt with lithium bis(trimethylsilyl)amide in THF/pyridine due to its superior solubility. As evidenced by ^1^H NMR and ^13^C NMR spectroscopy, the carbene **7** proved to be stable in pyridine-*d*_5_ solution up to 50 °C. The resonance frequency of the carbene carbon atom can be detected at δ = 203 ppm. Minute traces of water protonate the anionic NHC **7** spontaneously to give **6A/B** without any traces of decomposition products.

**Figure 2:**
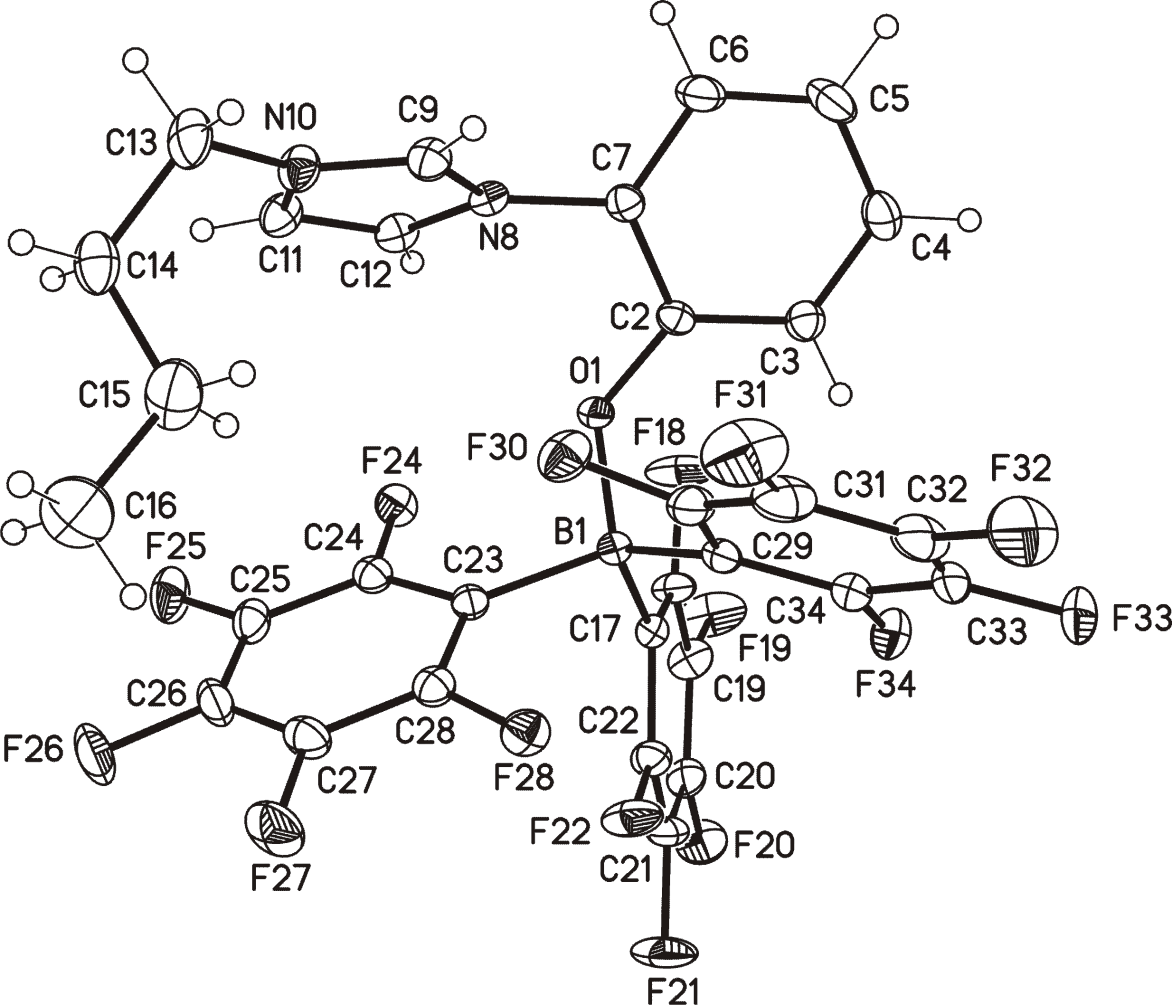
Molecular drawing of one of the two crystallographic independent molecules of borane adduct **8** (displacement parameters are drawn at 50% probability level; crystallographic numbering).

The betaine **6A** reacted with tris(pentafluorophenyl)borane in dioxane at reflux temperature to give the borane adduct **8**. The proton attached to C-2 of the imidazolium ring (crystallographic numbering, cf. Figure 2: C9) is clearly detectable at δ = 9.22 ppm in DMSO-*d*_6_, while the boron and fluorine atoms give resonance frequencies at δ = −3.45 ppm, and δ = −133.91, −159.97, −165.18 ppm in the ^11^B NMR and ^19^F NMR spectra, respectively. A single crystal X-ray analysis of the borane adduct displays two different conformers in the unit cell which differ inter alia in the torsion angles around the N_imidazole_–C_phenol_ bonds [−53.32(16)° vs 114.12(13)°] and the C_phenyl_–O bonds [49.43(17)° vs −15.29(18)°] (Figure 2).

**Figure 3:**
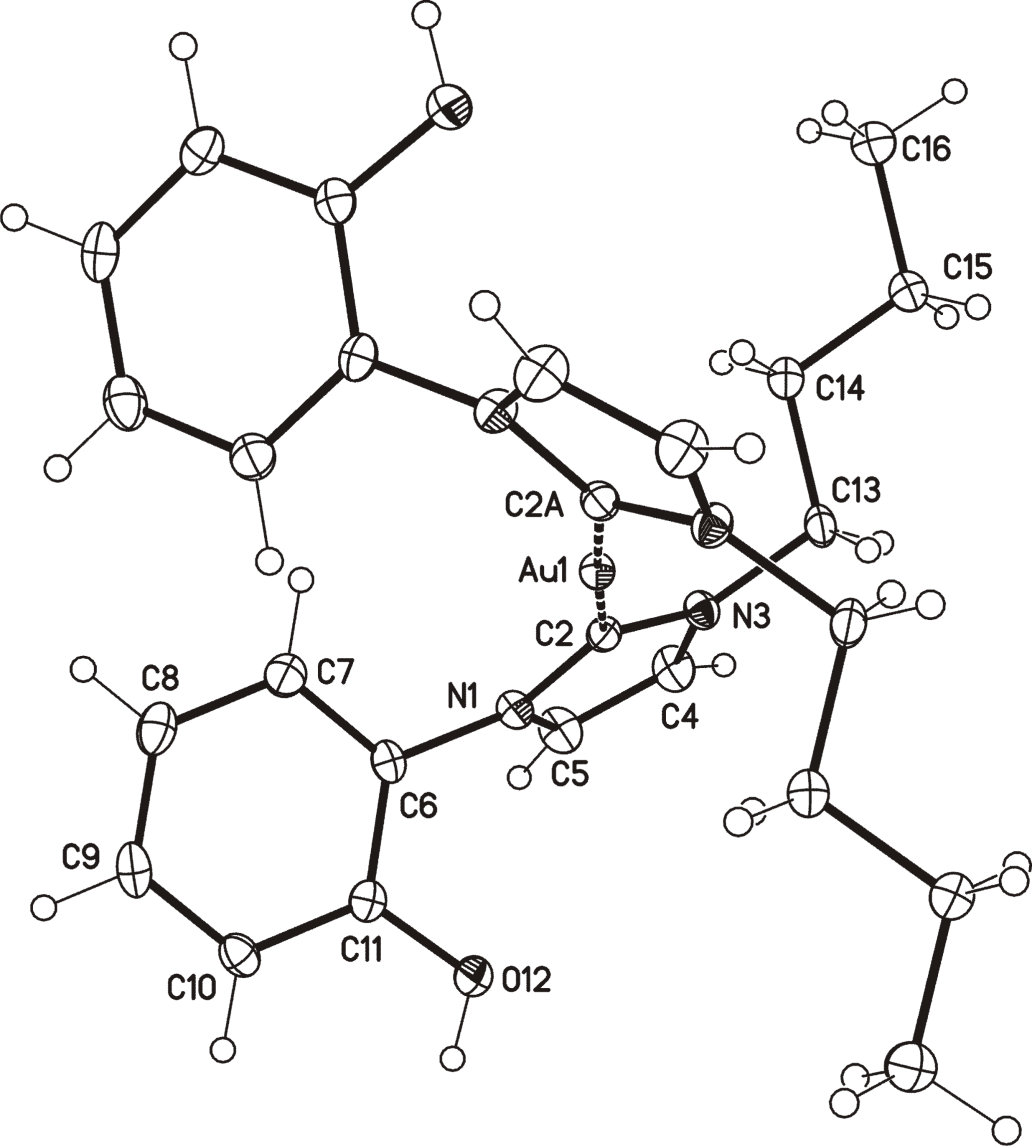
Molecular drawing of the cation of the gold complex **9** (displacement parameters are drawn at 50% probability level; crystallographic numbering).

A gold complex of the N-heterocyclic carbene tautomer **6B** can be obtained on reaction with (triphenylphosphine)gold(I) chloride in boiling anhydrous THF under a nitrogen atmosphere, under which conditions the colorless gold complex [Au(**6B**)_2_][Cl] (**9**) is formed in 60% yield (Scheme 1). The protons of the OH resonate at δ = 10.34 ppm in DMSO-*d*_6_, and the ^13^C NMR chemical shift of the carbene atom appears at δ = 183.9 ppm. Single crystals of the gold complex **9** were obtained by slow evaporation of a concentrated solution of **9** in MeOH and a molecular drawing is shown in Figure 3. The X-ray structure revealed a mixed occupation of the same position with chloride and bromide anions (4:1). The bond length between the gold atom and the carbene carbon atom (crystallographic numbering: Au1–C2) was found to be 202.11(15) pm and this value corresponds to the distances in other NHC gold complexes [36–37].

The coordination about the Au(I) center is almost linear, as a C2–Au(1)–C2A bond angle of 177.35(8)° was found. However, in contrast to other gold complexes, the two imidazole rings are not coplanar [38]. In addition, the imidazole and the phenol rings are twisted about −119.84(16)° (C5–N1–C6–C7) relative to the imidazole ring. Two Au complexes are connected via one halide anion which is involved in hydrogen bonds between two OH groups. On treatment with methanolic potassium carbonate, the two OH groups of gold complex **9** can be deprotonated to give the anionic complex [K][Au(**7**)_2_] **10** of the anionic N-heterocyclic carbene **7** in quantitative yield (Scheme 1). ESI mass spectra taken in the anion detection mode show the peak of **10** as base peak at *m*/*z* = 627.

**Scheme 2:**
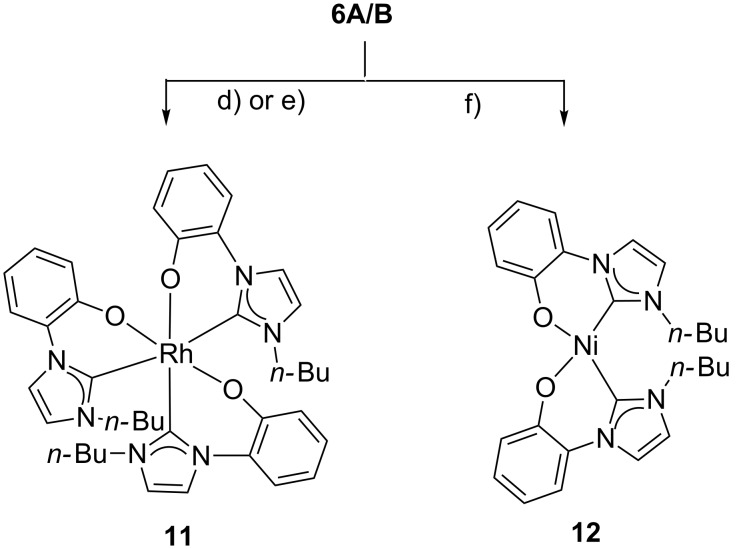
The anionic NHC **7** forms a rhodium complex and a nickel complex. d) [RhCl(COD)]_2_, toluene, rt to reflux (50%). e) [RhCl(PPh_3_)_2_(CO)], toluene, reflux, 57%. f) NiCl_2_(PPh_3_)_2_, THF, rt to reflux, 45%.

The anionic N-heterocyclic carbene **7** also forms a rhodium and a nickel complex (Scheme 2). The colorless rhodium complex [Rh(**7**)_3_] **11** was prepared on reaction of the tautomeric mixture **6A/B** with either chlorido(1,5-cyclooctadiene)rhodium(I) dimer, or with bis(triphenylphosphine)rhodium(I) carbonyl chloride in anhydrous toluene at reflux temperature, respectively. During these reactions, the water of crystallization of the starting material is – at least partially – removed by azeotropic distillation and Rh(I) is obviously oxidized to Rh(III). In order to balance the chemical equation, the formation of elemental hydrogen and of one equivalent of HCl can be postulated. Neither hydrogen nor reduced species such as cyclooctane or cyclooctene, however, have been detected. In the ESI mass spectra the base peak was detected at *m*/*z* = 749.2 which corresponds to the molecular peak of a [Rh(**7**)_3_ + H]^+^ complex. In the ^13^C NMR spectra, the C_carbene_ resonance frequencies were detected at 173.5 ppm, 171.0 ppm, and 164.4 ppm and have been identified by their ^1^*J*_RhCcarbene_ couplings of 35.6 Hz, 35.6 Hz, and 48.5 Hz, respectively. These chemical shifts are in a more upfield region and the coupling constants are smaller than in other complexes such as the neutral N-heterocyclic oxocarbene (NHOC) rhodium complex ([Rh(NHOC)Cl(COD)] of **2** (R = Mes; δ_Ccarbene_ = 229.7 ppm; ^1^*J*_RhCcarbene_ = 51.5 Hz) as well as its enol ethers (δ_Ccarbene_ = 171–177 ppm) [29,39], and other Rh complexes [40–41]. Correspondingly the phenol proton signals of the three geometrically non-equivalent ligands appear at different resonance frequencies. Thus, the three unsubstituted *ortho* positions of the phenolate moieties are detectable at 6.93–6.97 ppm (overlapped), 6.72 ppm, and 5.81 ppm, respectively.

**Figure 4:**
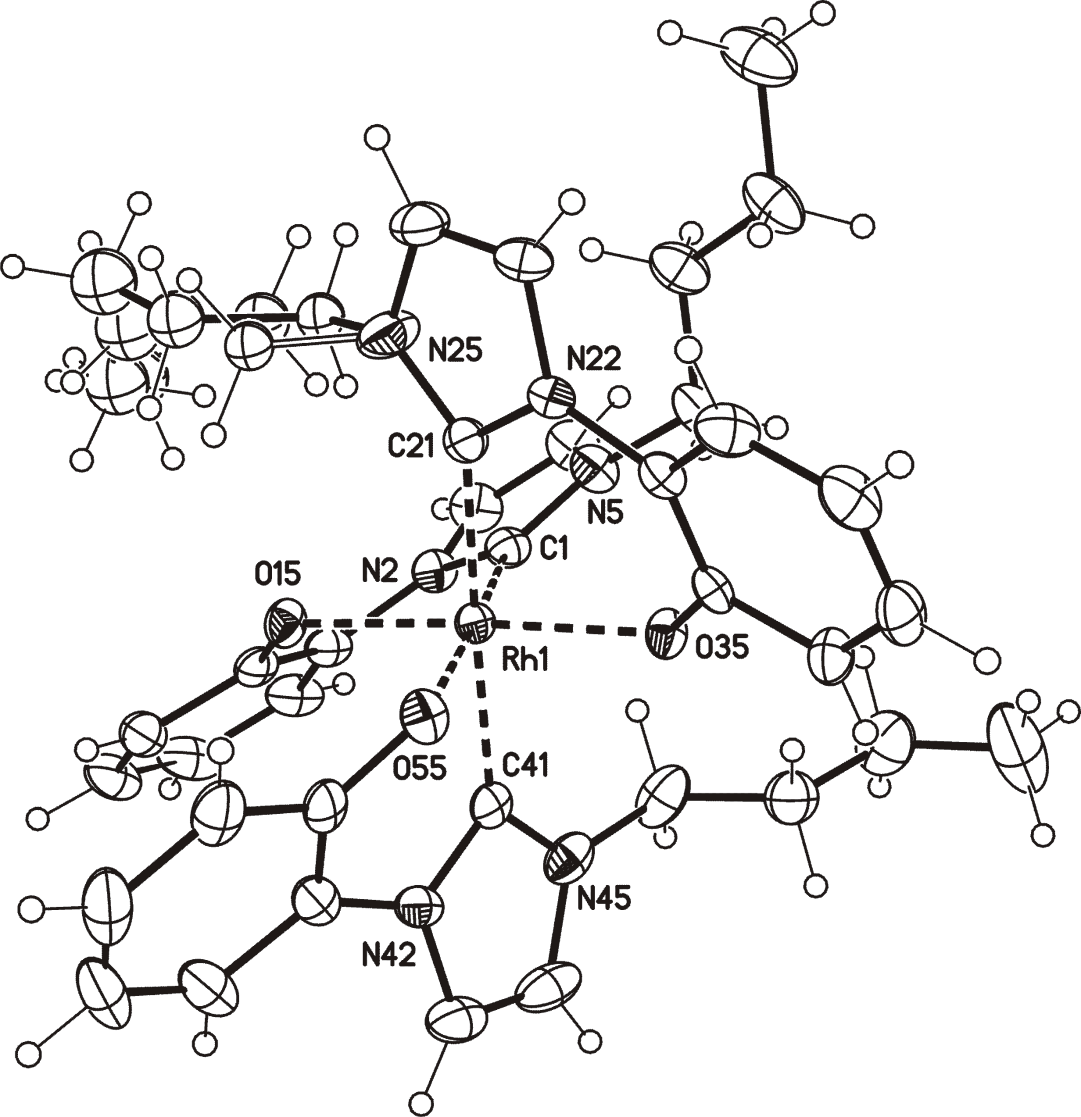
Molecular drawing of rhodium complex **11** (minor disorder parts omitted for clarity, displacement parameters are drawn at 50% probability level; crystallographic numberings).

Single crystals of [Rh(**7**)_3_] (**11**) were obtained by slow evaporation of a concentrated solution of **11** in a mixture of EtOAc and MeOH. The single crystal X-ray analysis proved that three anionic N-heterocyclic carbenes **7** serve as bidentate ligands, respectively (Figure 4). The *n*-butyl group connected to N25 is disordered. The bond lengths between the rhodium atom and the carbene atoms (C1, C21 and C41) were determined to be 196.6(2), 204.7(2) and 204.4(2) pm. In the literature-known complexes [Rh(ICy_3_)(CO)][PF_6_], [Rh(IiPr_2_Me_2_)_3_(CO)][PF_6_], and [Rh(ICy)(IiPr_2_Me_2_)_2_(CO)][PF_6_] bond lengths between 206.38(11) and 214.90(13) pm were found [40], whereas the two independent molecules of [Rh(IBioxMe_4_)_3_][BAr^F^_4_] in the elemental cell have one Rh–C_carbene_ bond of 194.1(2) pm and 193.4(2) pm, respectively, *trans* to a free coordination site of this naked low-coordinate rhodium complex [41]. Correspondingly the Rh–O15 (202.49(14) pm) and Rh–O35 bond lengths *cis* to the shortened Rh–C_carbene_ bond in **11** are identical (2.0244(14) pm), whereas the Rh–O55 bond *trans* to this bond is longer (209.98(15) pm). The phenolate rings are twisted in relation to the imidazole rings and the determined dihedral angles for C1–N2–C10–C15, C21–N22–C30–C35, and C41–N42–C50–C55 are 26.371(5)°, 18.939(5)°, and −33.008(5)°, respectively. The carbenes are twisted by approximately 28.1° to 37.0° in relation to the Rh–C_carbene_ bonds.

**Figure 5:**
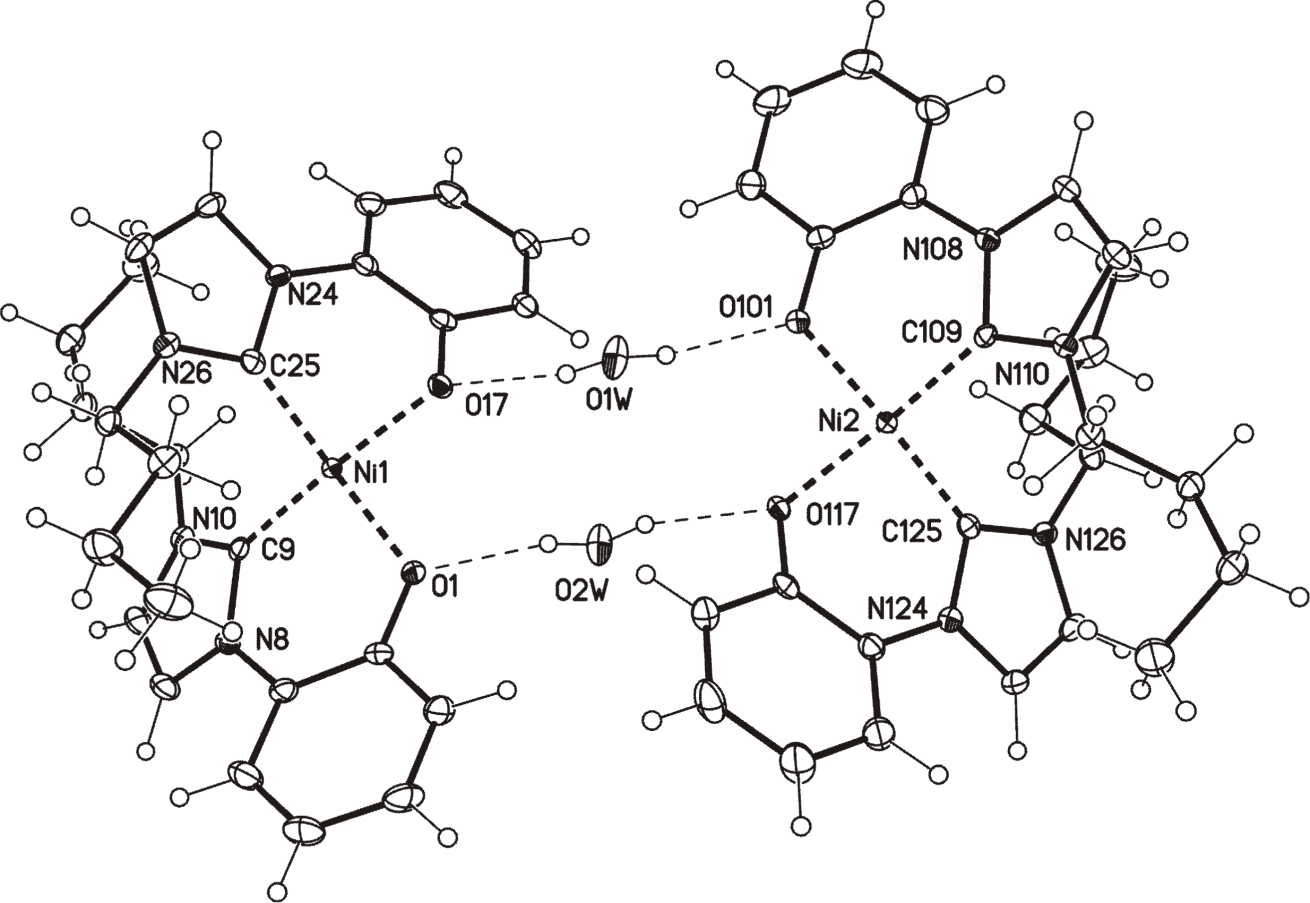
Molecular drawing of the dimeric nickel complex **12** (displacement parameters are drawn at 50% probability level; crystallographic numberings).

The reaction of **6A/6B** with bis(triphenylphosphine)nickel(II) chloride at reflux temperature resulted in the formation of the nickel complex [Ni(**7**)_2_] (**12**, Scheme 2). Single crystals were obtained by slow evaporation of **12** from EtOAc/MeOH. The X-ray analysis shows two independent nickel complexes which are connected via hydrogen bonds to two water molecules (Figure 5). The nickel atom is in the center of an essentially square planar environment, as the sums of the bond angles are 363.81° and 364.76°, respectively. The ligand adopts a *cis* arrangement around the Ni atom. The Ni–O bond lengths were determined to be 187.60(13) pm and 190.11(13) pm, whereas the Ni–C_carbene_ bonds have lengths between 184.06(19) pm and 184.82(19) pm, respectively. These values correspond to literature-known bond lengths of *cis* arranged bidentate ligands around Ni [42–44], but, as expected, they differ from those of [(PEt_3_)(Ph)Ni(imidazo[1,5-*a*]quinolin-9-olate-1-ylidene)] [45] as well as Ni complexes with tridentate ligands [46–47].

## Conclusion

In summary, this publication presents four molecules which differ only in their number and positions of protons. Starting from cation **5**, the neutral compound **6A** (betaine) was formed and detected spectroscopically, whereas its tautomeric carbene **6B** could not be characterized in non-complexed form. Deprotonation of **6A/B** gave the anionic NHC **7**. The two tautomers **6A/6B** and the anionic NHC **7** could be trapped as complexes. These results supplement our knowledge about the interesting area of overlap between mesomeric betaines and N-heterocyclic carbenes [34] and of structurally related zwitterionic motifs known in the literature [48–52].

## Experimental

**General considerations.** Silica gel 60 (0.040–0.063 mm) was used for flash-chromatographic separations. The NMR spectra were obtained with a Bruker Avance 400 and Bruker Avance III 600 MHz spectrometer, and ^1^H NMR spectra were recorded at 400 MHz or 600 MHz. ^13^C NMR spectra were recorded at 100 MHz or 150 MHz. The solvent peak or tetramethylsilane was used as the internal reference. The multiplicities are described here by using the following abbreviations: s = singlet, d = doublet, t = triplet, q = quartet, and m = multiplet, and the signal orientations in DEPT experiments were described as follows: o = no signal; + = up (CH, CH_3_); − = down (CH_2_). For NMR peak assignments we numbered the compounds not necessarily in accordance with IUPAC rules to allow comparisons. Thus, “C-2” always refers to C-2 of the imidazolium and imidazol-2-ylidene ring, respectively. The spectroscopic numbering, which differ from the crystallographic numbering, is presented on the NMR spectra in Supporting Information File 1. All FTIR spectra were measured with a Bruker Alpha T spectrometer in the range of 400 to 4000 cm^−1^. The mass spectra were obtained with a Varian 320 MS Triple Quad GC/MS/MS instrument with a Varian 450-GC. All electrospray ionisation mass spectra (ESIMS) were measured with a Hewlett-Packard/Agilent LCMSD series HP 1100 spectrometer with APIES. The compound samples were sprayed from MeOH at 4000 V capillary voltage and fragmentor voltages of 30 V, unless otherwise noted. Melting points are uncorrected and were determined in an apparatus according to Dr. Tottoli (Büchi). All HRMS spectra were obtained with a Bruker Daltonik Tesla-Fourier transform–ion cyclotron resonance mass spectrometer with electrospray ionisation.

The single-crystal X-ray diffraction studies were carried out on a Bruker D8 Venture diffractometer with Photon100 detector at 123(2) K using Cu Kα (**8**) and Mo Kα radiation (**9**, **11**, **12**) (λ = 1.54178 Å and 0.71073 Å). Dual space methods (**8**, SHELXT) [53] and direct methods (**9**, **11**, **12**, SHELXS-97) [54] were used for structure solution and refinement was carried out using SHELXL-2014 (full-matrix least-squares on F2). Hydrogen atoms were localized by difference electron density determination and refined using a riding model (H(O) free). Semi-empirical absorption corrections were applied. For **8**, **9**, and **11** an extinction correction was applied. In **9** the halogen counter anion (occupancy Cl/Br approx. 81:19) and in **11** one *n*-butyl group are disordered.

**8:** colorless crystals, C_31_H_16_BF_15_N_2_O, *M*_r_ = 728.27, crystal size 0.35 × 0.25 × 0.15 mm, monoclinic, space group *P*2_1_/*n* (No. 14), *a* = 21.9006(7) Å, *b* = 13.1620(4) Å, *c* = 22.0149(7) Å, β = 117.982(1)°, *V* = 5650.1(3) Å^3^, *Z* = 8, ρ = 1.712 Mg/m^−3^, µ(Cu Kα) = 1.552 mm^−1^, F(000) = 2912, 2Θ_max_ = 144.4°, 56172 reflections, of which 11099 were independent (*R*_int_ = 0.024), 902 parameters, *R*_1_ = 0.030 (for 10343 I > 2σ(I)), w*R*_2_ = 0.074 (all data), S = 1.04, largest diff. peak/hole = 0.359/−0.191 e Å^−3^. There are 2 different conformers in the asymmetric unit.

**9:** colorless crystals, C_26_H_32_AuN_4_O_2_∙0.19(Br)∙0.81(Cl), *M*_r_ = 673.42, crystal size 0.24 × 0.12 × 0.06 mm, monoclinic, space group *C*2/*c* (No. 15), *a* = 15.0275(9) Å, *b* = 13.1392(7) Å, *c* = 13.0205(7) Å, β = 104.161(2)°, *V* = 2492.8(2) Å^3^, *Z* = 4, ρ = 1.794 Mg/m^−3^, µ(Mo Kα) = 6.324 mm^−1^, F(000) = 1326, 2Θ_max_ = 55.2°, 46420 reflections, of which 2867 were independent (*R*_int_ = 0.026), 162 parameters, *R*_1_ = 0.011 (for 2788 I > 2σ(I)), w*R*_2_ = 0.026 (all data), *S* = 1.10, largest diff. peak/hole = 0.404/−0.489 e Å^−3^, disorder of the halogen counter anion (occupancy Cl/Br 0.813(2):0.187(2)).

**11:** yellow crystals, C_39_H_45_N_6_O_3_Rh, *M*_r_ = 748.72, crystal size 0.38 × 0.32 × 0.16 mm, monoclinic, space group *P*2_1_/*n* (No. 14), *a* = 11.7692(5) Å, *b* = 18.8111(7) Å, *c* = 16.2459(7) Å, β = 104.615(2)°, *V* = 3480.3(2) Å^3^, *Z* = 4, ρ = 1.429 Mg/m^−3^, µ(Mo Kα) = 0.538 mm^−1^, F(000) = 1560, 2Θ_max_ = 55.2°, 90530 reflections, of which 8005 were independent (*R*_int_ = 0.046), 436 parameters, 146 restraints, *R*_1_ = 0.032 (for 6902 I > 2σ(I)), w*R*_2_ = 0.071 (all data), *S* = 1.05, largest diff. peak/hole = 0.645/−0.996 e Å^−3^, disorder of the *n*-butyl group.

**12:** yellow crystals, C_26_H_30_N_4_NiO_2_∙H_2_O, *M*_r_ = 507.26, crystal size 0.22 × 0.12 × 0.04 mm, monoclinic, space group *P*2_1_/*n* (No. 14), *a* = 11.2267(5) Å, *b* = 22.8310(9) Å, *c* = 19.1274(8) Å, β = 100.099(2)°, *V* = 4826.7(4) Å^3^, *Z* = 8, ρ = 1.396 Mg/m^−3^, µ(Mo Kα) = 0.839 mm^−1^, F(000) = 2144, 2Θ_max_ = 55.2°, 89393 reflections, of which 11117 were independent (*R*_int_ = 0.070), 625 parameters, 6 restraints, *R*_1_ = 0.038 (for 8729 I > 2σ(I)), w*R*_2_ = 0.095 (all data), *S* = 1.06, largest diff. peak/hole = 1.063/−0.370 e Å^−3^, dimer.

CCDC 1489189 (**8**), 1489190 (**9**), 1489191 (**11**), and 1489192 (**12**) contain the supplementary crystallographic data for this paper. These data can be obtained free of charge from The Cambridge Crystallographic Data Centre via http://www.ccdc.cam.ac.uk/data_request/cif.

**Preparation of lithium 2-(3-butyl-1*H*-imidazol-2-ylidene-1-yl)phenolate (7):** A solution of 0.02 g (0.09 mmol) of 2-(3-butyl-1*H*-imidazolium-1-yl)phenolate [35] and 0.10 mL of lithium bis(trimethylsilyl)amide (1.0 M solution in THF) in 0.7 mL of pyridine was stirred for 30 minutes at rt. The product was characterized in solution, as traces of moisture reconstituted the starting material. Concentration of the solution in vacuo, filtration and crystallization of the mother liquor in the presence of one drop of water gave **6A/B** in quantitative yield. Yield of **7**: 0.02 g (100%). ^1^H NMR (600 MHz, pyridine-*d*_5_) δ 7.44 (d, *J* = 0.8 Hz, 1H, H-5), 7.40 (dd, *J*_1_ = 1.0 Hz, *J*_2_ = 7.6 Hz, 1H, H-11), 7.16–7.12 (m, 2H, H-9/8), 7.05 (d, *J* = 0.8 Hz, 1H, H-4), 6.59 (ddd, *J*_1_ = 0.9 Hz, *J*_2_ = 7.3 Hz, *J*_3_ = 7.6 Hz, 1H, H-10), 3.94 (t, *J* = 7.0 Hz, 2H, H-12), 1.68–1.62 (m, 2H, H-13), 1.19–1.15 (m, 2H, H-14), 0.75 (t, *J* = 7.3 Hz, 3H, H-15) ppm; ^13^C NMR (150 MHz, pyridine-*d*_5_) δ 203.1 (o, C-2), 162.2 (o, C-7), 131.0 (o, C-6), 127.2 (+, C-9), 123.0 (+, C-8), 122.7 (+, C-11), 119.5 (+, C-5), 118.5 (+, C-4), 111. 2 (+, C-10), 50.7 (−, C-12), 33.5 (−, C-13), 19.8 (−, C-14), 13.5 (+, C-15) ppm; ESIMS (50 V) *m*/*z* (%): 215.1 (100) [M − Li]^−^.

**Preparation of (2-(3-butyl-1*H*-imidazolium-1-yl)phenoxy)- tris(perfluorophenyl)borate (8):** Under an inert atmosphere a solution of 0.108 g (0.50 mmol) of 2-(3-butyl-1*H*-imidazolium-1-yl)phenolate and 0.152 mg (1.0 mmol) of tris(pentafluorophenyl)borane in 10 mL of dry dioxane was stirred at reflux for 4 h in a bomb tube. Then, the solvent was distilled off in vacuo. The product was separated by column chromatography (silica gel, EtOAc). Yield: 0.155 g (43%) of a colorless solid, mp 230 °C; ^1^H NMR (600 MHz, DMSO-*d*_6_) δ 9.22 (dd, *J*_1_ = 1.4 Hz, *J*_2_ = 1.5 Hz, 1H, H-2), 7.90 (dd, *J*_1_ = 1.4 Hz, *J*_2_ = 1.5 Hz, 1H, H-4), 7.82 (dd, *J*_1_ = 1.4 Hz, *J*_2_ = 1.5 Hz, 1H, H-5), 7.43 (dd, *J*_1_ = 2.3 Hz, *J*_2_ = 9.8 Hz, 1H, H-11), 7.17 (ddd, *J*_1_ = 2.3 Hz, *J*_2_ = 7.4 Hz, *J*_3_ = 8.4 Hz, 1H, H-9), 6.81 (ddd, *J*_1_ = 1.0 Hz, *J*_2_ = 7.4 Hz, *J*_3_ = 9.8 Hz, 1H, H-10), 6.60 (dd, *J*_1_ = 1.0 Hz, *J*_2_ = 8.4 Hz, 1H, H-8), 4.18 (t, *J* = 10.8 Hz, 2H, H-12), 1.72–1.64 (overlapped, 2H, H-13), 1.22–1.13 (overlapped, 2H, H-14), 0.84 (t, *J* = 11.0 Hz, 3H, H-15) ppm; ^13^C NMR (150 MHz, DMSO-*d*_6_) δ 152.5 (o, C-7), 147.2 (o, d, ^1^*J*_C,F_ = 242.1 Hz, C-20), 138.2 (o, d, ^1^*J*_C,F_ = 230.3 Hz, C-21), 136.3 (+, C-2), 135.8 (o, d, ^1^*J*_C,F_ = 235.5 Hz, C-19), 130.2 (+, C-9), 125.4 (o, C-6), 125.0 (+, C-11), 123.5 (+, C-5), 121.8 (o, C-4), 122.2–121.3 (+/o, C-18), 117.7 (+, C-10), 117.4 (+, C-8), 48.7 (−,C-12), 31.4 (−, C-13), 18.6 (−, C-14), 13.0 (+, C-15) ppm; ^11^B NMR (DMSO-*d*_6_, 128 MHz, external reference) δ −3.45 ppm; ^19^F NMR (DMSO-*d*_6_, 565 MHz, Cl_3_CF) δ −133.91 (d, ^3^*J*_F,F_ = 21.5 Hz, 6F, F-19/19'), −159.97 (t, ^3^*J*_F,F_ = 21.5 Hz, 3F, F-21), −165.18 (dd, ^3^*J*_F,F_ = 21.5 Hz, 6F, F-20/20', partially overlapped) ppm; IR (ATR) 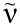: 1511, 1498, 1456, 1304, 1277, 1262, 1079, 1038, 974, 965, 945, 936, 802, 768, 762, 755, 733, 691, 673, 667, 654, 649 cm^−1^; ESIMS (50 V) *m/z* (%): 727.0 (100) [M − H]^−^. HRESIMS: calcd for C_31_H_15_N_2_OF_15_B^−^, 727.1038; found, 727.1038.

**Preparation of bis(3-butyl-1-(2-hydroxyphenyl)-1*H*-imidazolium-2-yl)gold monochloride (9):** A solution of 0.43 g (0.20 mmol) of 2-(3-butyl-1*H*-imidazolium-1-yl)phenolate in 5 mL of anhydrous THF was treated with 0.05 g (0.10 mmol) of (triphenylphosphine)gold(I) chloride and stirred under an inert atmosphere overnight at reflux temperature. The resulting solid was filtered off, washed with THF, and dried in vacuo. Yield: 0.40 g (60%) of a yellow solid, mp 242 °C; ^1^H NMR (600 MHz, CD_3_OD) δ 7.39 (d, *J* = 2.0 Hz, 2H, H-4/4'), 7.37 (dd, *J*_1_ = 1.6 Hz, *J*_2_ = 7.7 Hz, 2H, H-11/11'), 7.38–7.33 (overlapped signals, 4H, H-5/5'/9/9'), 7.01 (dd, *J*_1_ = 1.3 Hz, *J*_2_ = 8.2 Hz, 2H, H-8/8'), 6.93 (ddd, *J*_1_ = 1.3 Hz, *J*_2_ = 7.5 Hz, *J*_3_ = 7.7 Hz, 2H, H-10/10´), 4.01 (t, *J* = 7.1 Hz, 4H, H-12/12'), 1.71–1.66 (m, 4H, H-13/13'), 1.22–1.15 (m, 4H, H-14/14'), 0.88 (t, *J* = 7.3 Hz, 6H, H-15/15') ppm; ^1^H NMR (600 MHz, DMSO-*d*_6_) δ 10.34 (s broad, 2H, OH), 7.62 (d, *J* = 1.9 Hz, 2H, H-2/2'), 7.57 (d, *J* = 1.9 Hz, 2H, H-3/3'), 7.38 (dd, *J*_1_ = 1.7 Hz, *J*_2_ = 7.8 Hz, 2H, H-11/11'), 7.34 (ddd, *J*_1_ = 1.7 Hz, *J*_2_ = 7.6 Hz, *J*_3_ = 8.2 Hz, 2H, H-9/9'), 7.06 (dd, *J*_1_ = 1.3 Hz, *J*_2_ = 8.2 Hz, 2H, H-8/8'), 6.93 (ddd, *J*_1_ = 1.3 Hz, *J*_2_ = 7.6 Hz, *J*_3_ = 7.8 Hz, 2H, H-10/10'), 3.96 (t, *J* = 7.0 Hz, 4H, H-12/12'), 1.59–1.64 (m, 4H, H-13/13'), 1.05–1.11 (m, 4H, H-14/14'), 0.81 (t, *J* = 7.3 Hz, 6H, H-15/15') ppm; ^13^C NMR (150 MHz, CD_3_OD) δ183.9 (o, C-2/2'), 152.5 (o, C-7/7'), 130.3 (+, C-9/9'), 128.1 (+, C-11/11'), 126.8 (o, C-6/6'), 123.8 (+, C-5/5'), 120.7 (+, C-4/4'), 119.1 (+, C-10/10'), 116.5 (+, C-8/8'), 50.4 (−, C-12/12'), 32.9 (−, C-13/13'), 19.2 (−, C-14/14'), 13.6 (+, C-15/15') ppm; IR (ATR) 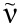: 2956, 1598, 1509, 1463, 1455, 1285, 1241, 829, 766, 752, 734, 687 cm^−1^; MS (ESI 5 V) *m/z* (%) = 629.1 (100) M^+^. HRESIMS: calcd for C_26_H_32_N_4_O_2_Au^+^, 629.2192; found, 629.2191.

**Preparation of potassium bis(3-butyl-1-(2-phenolate)-1*H*-imidazolium-2-yl)gold (10)**: A solution of 0.066 g (0.10 mmol) of bis(3-butyl-1-(2-hydroxyphenyl)-1*H*-imidazolium-2-yl)gold chloride (**9**) and 0.014 g (0.10 mmol) of K_2_CO_3_ in 5 mL of methanol was stirred for 30 minutes under ultrasound irradiation. After concentrating the solution in vacuo, the resulting colorless solid was filtered off. Yield: 0.066 g (100%), mp 240 °C; ^1^H NMR (600 MHz, CD_3_OD) δ 7.34 (d, *J* = 1.8 Hz, 2H, H-4/4'), 7.21–7.23 (overlapped signals, 4H, H-4/4' and H-11/11'), 7.08 (ddd, *J*_1_ = 1.86 Hz, *J*_2_ = 7.3 Hz, *J*_3_ = 8.3 Hz, 2H, H-9/9'), 6.80 (dd, *J*_1_ = 1.2 Hz, *J*_2_ = 8.3 Hz, 2H, H-8/8'), 6.43 (ddd, *J*_1_ = 1.2 Hz, *J*_2_ = 7.3 Hz, *J*_3_ = 7.5 Hz, 2H, H-10/10'), 4.00 (t, *J* = 7.1 Hz, 4H, H-12/12'), 1.70–1.65 (m, 4H, H-13/13'), 1.26–1.19 (m, 4H, H-14/14'), 0.88 (t, *J* = 7.3 Hz, 6H, H15/15') ppm; ^13^C NMR (150 MHz, CD_3_OD) δ 183.5 (o, C-2/2'), 162.5 (o, C-7), 160.1(o, C-7'), 129.5 (+, C-9/9'), 129.2 (o, C-6/6'), 127.4 (+, C-11/11'), 124.1 (+, C-5/5'), 121.2 (+, C-8/8'), 119.7 (+, C-4/4'), 111.9 (+, C-10/10'), 50.2 (−, C-12/12'), 33.1 (−, C-13/13'), 19.3 (−, C-14/14'), 12.7 (+, C-15/15') ppm; IR (ATR) 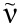: 3157, 2958, 1637, 1591, 1483, 1447, 1311, 1246, 1076, 1061, 844, 748, 701, 566 cm^−1^; ESIMS (50 V) *m*/*z* (%): 627.2 (100) [M − K]^−^.

**Preparation of tris(3-butyl-1-(2-oxidophenyl)-1*H*-imidazolium-2-yl)rhodium (11):** Method A: A solution of 0.16 g (0.72 mmol) of 2-(3-butyl-1*H*-imidazolium-1-yl)phenolate in 5 mL of anhydrous toluene was treated with 0.06 g (0.12 mmol) of chloro(1,5-cyclooctadiene)rhodium(I) dimer and stirred under an inert atmosphere overnight at reflux temperature. The resulting colorless solid was filtered off, washed with THF and dried in vacuo. Yield: 0.09g (50%) of a yellow solid, mp: 280 °C. Method B: A solution of 0.16 g (0.72 mmol) of 2-(3-butyl-1*H*-imidazolium-1-yl)phenolate in 5 mL of anhydrous toluene was treated with 0.17 g (0.24 mmol) of bis(triphenylphosphine)rhodium(I) carbonyl chloride and stirred under an inert atmosphere overnight at reflux temperature. The resulting colorless solid was filtered off, washed with THF and dried in vacuo. Yield: 0.10g (57%) of a yellow solid, mp 280 °C; ^1^H NMR (600 MHz, CD_3_OD) δ 8.00 (d, *J* = 2.2 Hz, 1H, H-5), 7.82 (d, *J* = 2.2 Hz, 1H, H-5'), 7.52 dd, *J*_1_ = 1.5 Hz, *J*_2_ = 8.2 Hz, 1H, H-11), 7.44 (d, *J* = 2.2 Hz, 1H, H-4), 7.39 (d, *J* = 2.0 Hz, 1H, H-4'), 7.29 (d, *J* = 2.2 Hz, 1H, H-5''), 7.12 (d, *J* = 2.0 Hz, 1H, H-5'), 7.11 (dd, *J*_1_ = 1.5 Hz, *J*_2_ = 8.2 Hz, 1H, H-11'), 7.05 (dd, *J*_1_ = 1.4 Hz, *J*_2_ = 8.1 Hz, 1H, H-11''), 6.97–6.93 (m, 2H, H-8/9), 6.80 (ddd, *J*_1_ = 1.5 Hz, *J*_2_ = 7.0 Hz, *J*_3_ = 8.2 Hz, 1H, H-9'), 6.72 (dd, *J*_1_ = 1.5 Hz, *J*_2_ = 8.3 Hz, 1H, H-8'), 6.64–6.60 (overlapped signals, 2H, H-9''/10), 6.57 (ddd, *J*_1_ = 1.5 Hz, *J*_2_ = 7.0 Hz, *J*_3_ = 8.3 Hz, 1H, H-10'), 6.41 (ddd, *J*_1_ = 1.4 Hz, *J*_2_ = 7.3 Hz, *J*_3_ = 8.1 Hz, 1H , H-10''), 5.81 (dd, *J*_1_ = 1.3 Hz, *J*_2_ = 8.1 Hz, 1H, H-8''), 4.33 (ddd, *J*_1_ = 4.8 Hz, *J*_2_ = 11.8 Hz, *J*_3_ = 16.7 Hz, 1H, H-12), 4.49 (ddd, *J*_1_ = 4.8 Hz, *J*_2_ = 11.8 Hz, *J*_3_ = 16.7 Hz, 1H, H12), 3.78 (t, *J* = 8.5 Hz, 2H, H-12'), 3.78–3.64 (m, 2H, H-12''), 1.70–1.64 (m, 1H, H-13), 1.62–1.56 (m, 1H, H-13), 1.53–1.45 (m, 1H, H-13'), 1.37–1.30 (m, 1H, H-13'), 1.27–1.18 (m, 1H, H-14), 1.08–0.89 (m, 8H, 13''/14/ 2 × H-14'/14''/15), 0.88–0.82 (m, 1H, H-14'), 0.79–0.72 (m, 1H, H-13''), 0.68 (t, *J* = 7.3 Hz, 3H, H-15'), 0.63 (t, *J* = 7.4 Hz, 3H, H-15'') ppm; ^13^C NMR (150 MHz, CD_3_OD) δ 173.5 (o, d, ^1^*J*_C,Rh_ = 35.6 Hz, C-2), 171.0 (o, d, ^1^*J*_C,Rh_ = 35.6 Hz, C-2'), 164.4 (o, d, ^1^*J*_C,Rh_ = 48.5 Hz, C-2''), 160.2 (o, C-7), 159.8(o, C-7'), 157.3 (o, C-7''), 130.5 (o, C-6), 129.8 (o, C-6'), 128.2 (o, C-6''), 126.6 (+, C-9), 126.1 (+, C-9'), 125.9 (+, C-9''), 123.4 (+, C-8), 122.9 (+, C-4), 122.7 (+, C-8'), 122.2 (+, C-4'), 121.3 (+, C-8''), 120.8 (+, C-4''), 120.7 (+, C-11), 119.6 (+, C-11'), 118.7 (+, C-11''), 118.4 (+, C-5), 118.4 (+, C-5'), 117.8 (+, C-5''), 115.1 (+, C-10), 114.1 (+, C-10'), 113.6 (+, C-10''), 48.7/48.3/48.2 (−, C-12/12'/12''), 32.5/32.3/32.0 (−, C-13/13'/13''), 20.0/19.7/19.7 (−, C-14/14'/14''), 12.9/12.4/12.4 (+, C-15/15'/15'') ppm; IR (ATR) 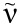: 2955, 1590, 1419, 1374, 1302, 1268, 1122, 950, 850, 742, 716, 698, 690, 683, 657 cm^−1^; ESIMS (30 V) *m*/*z* (%): 749.2 (100) [M+H]^+^; HRESIMS: calcd for C_39_H_46_N_6_O_3_Rh^+^, 749.2686; found, 749.2685.

**Preparation of bis(3-butyl-1-(2-oxidophenyl)-1*H*-imidazolium-2-yl)nickel (12):** A solution of 0.15 g (0.70 mmol) of 2-(3-butyl-1*H*-imidazolium-1-yl)phenolate in 5 mL of anhydrous toluene was treated with 0.23 g (0.35 mmol) of bis(triphenylphosphine)nickel(II) dichloride and stirred under an inert atmosphere overnight at reflux temperature. The resulting white solid was filtered off, washed with THF and dried in vacuo. Yield: 0.09 g (45%) of a yellow solid, mp: 153 °C; ^1^H NMR (600 MHz, CD_3_OD) δ 7.67 (d, *J* = 2.0 Hz, 2H, H-4/4'), 7.44 (dd, *J*_1_ = 1.3 Hz, *J*_2_ = 7.5 Hz, 2H, H-11/11'), 7.36 (d, *J* = 2.0 Hz, 2H, H-5/5'), 7.11 (dd, *J*_1_ = 1.9 Hz, *J*_2_ = 8.2 Hz, 2H, H-8/8'), 7.08 (ddd, *J*_1_ = 1.3 Hz, *J*_2_ = 8.2 Hz, *J*_3_ = 8.9 Hz, 2H, H-9/9'), 6.72 (ddd, *J*_1_ = 1.9 Hz, *J*_2_ = 7.5 Hz, *J*_3_ = 8.9 Hz, 2H, H-10/10'), 3.68–3.63 (m, 2H, H-12/12'), 3.06–3.01 (m, 2H, H-12/12'), 2.42–2.35 (m, 2H, H-13/13'), 1.84–1.76 (m, 2H, H-13/13'), 1.27–1.21 (m, 4H, H-14/14'), 0.77 (t, *J*_1_ = 7.4 Hz, 6H, H-15/15') ppm; ^13^C NMR (600 MHz, CD_3_OD) δ 156.8 (o, C-7/7'), 156.2 (o, C-2/2'), 128.8 (o, C-6/6'), 127.4 (+, C-9/9'), 124.2 (+, C-5/5'), 120.9 (+, C-8/8'), 118.4 (+, C-11/11'), 118.3 (+, C-4/4'), 115.1 (+, C-10/10'), 49.8 (+, C-12/12'), 33.4 (+, C-13/13'), 19.5 (+, C-14/14'), 12.3 (+, C-15/15') ppm; IR (ATR) 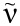: 2958, 2929, 2872, 1593, 1487, 1457, 1417, 1395, 1300, 1273, 1235, 1154, 952, 840, 742, 724, 681 cm^−1^; ESIMS (5 V) *m*/*z* (%): 511.0 (100) [M + Na]^+^; HRESIMS: calcd for C_26_H_31_N_4_O_2_Ni^+^; 489.1800; found, 489.1800.

## Supporting Information

File 1 NMR spectra and molecular drawings.
