# Supplementary material for: From betaines to anionic N-heterocyclic carbenes. Borane, gold, rhodium, and nickel complexes starting from an imidazoliumphenolate and its carbene tautomer
Source: Beilstein J Org Chem. 2016 Dec 8;12:2673–81. doi: 10.3762/bjoc.12.264 (PMC5238575; doi:10.3762/bjoc.12.264)
Supplement: File 1 — NMR spectra and molecular drawings. [file Beilstein_J_Org_Chem-12-2673-s001.pdf]

Supporting Information

for

From betaines to anionic N-heterocyclic carbenes. Borane, gold, rhodium, and nickel complexes starting from an imidazoliumphenolate and its carbene tautomer

Ming Liu[1], Jan C. Namyslo[1], Martin Nieger[2], Mika Polamo[2] and Andreas Schmidt*[1]

Address: [1]Clausthal University of Technology, Institute of Organic Chemistry, Leibnizstrasse 6, D-38678 Clausthal-Zellerfeld, Germany and [2]University of Helsinki, Department of Chemistry, P.O. Box 55, FIN-00014 Helsinki, Finland

Email: Andreas Schmidt - schmidt@ioc.tu-clausthal.de

*Corresponding author

NMR spectra and molecular drawings

Lithium 2-(3-butyl-1*H*-imidazol-2-ylidene-1-yl)phenolate (7)

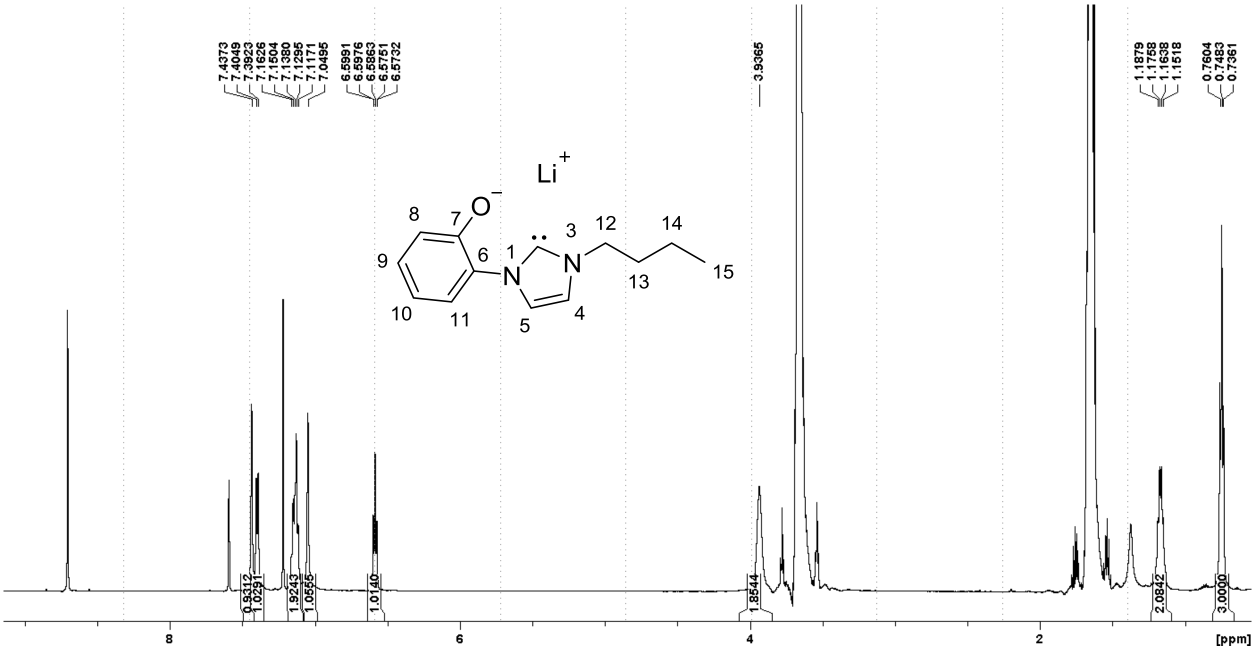

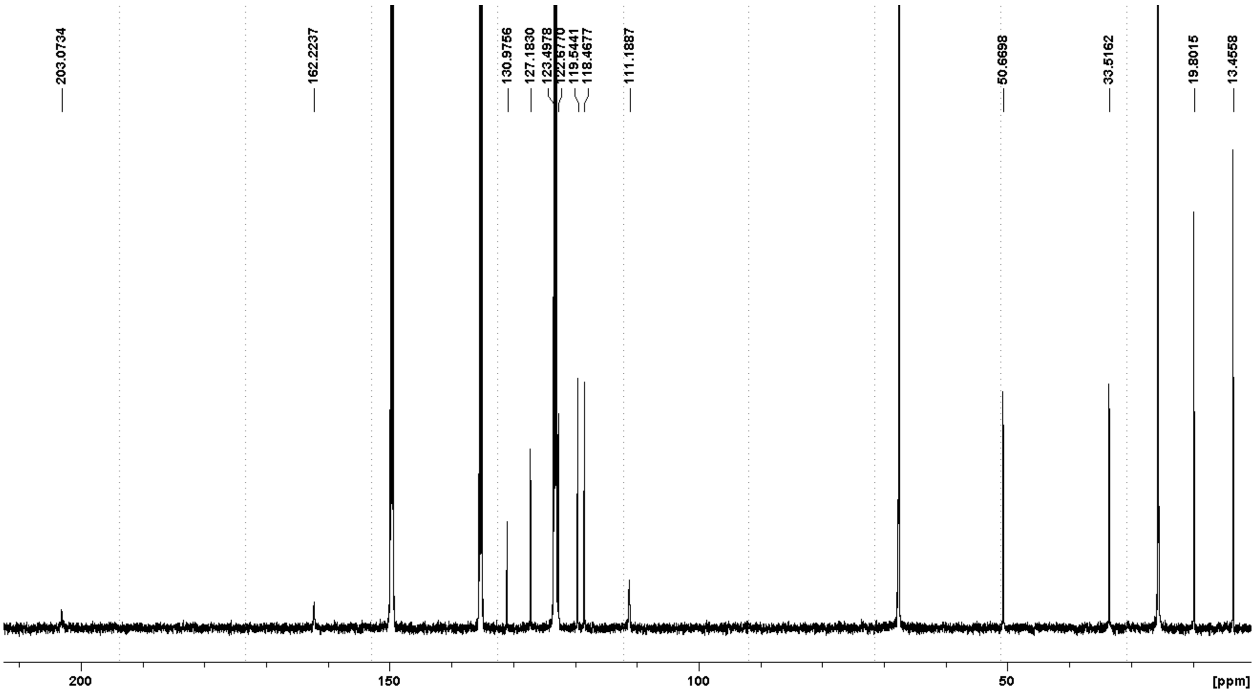

Figures S1/2: ^{1}H and ^{13}C NMR spectra of the lithium salt of the anionic NHC **7**.

(2-(3-Butyl-1*H*-imidazolium-1-yl)phenoxy)tris(perfluorophenyl)borate (8)

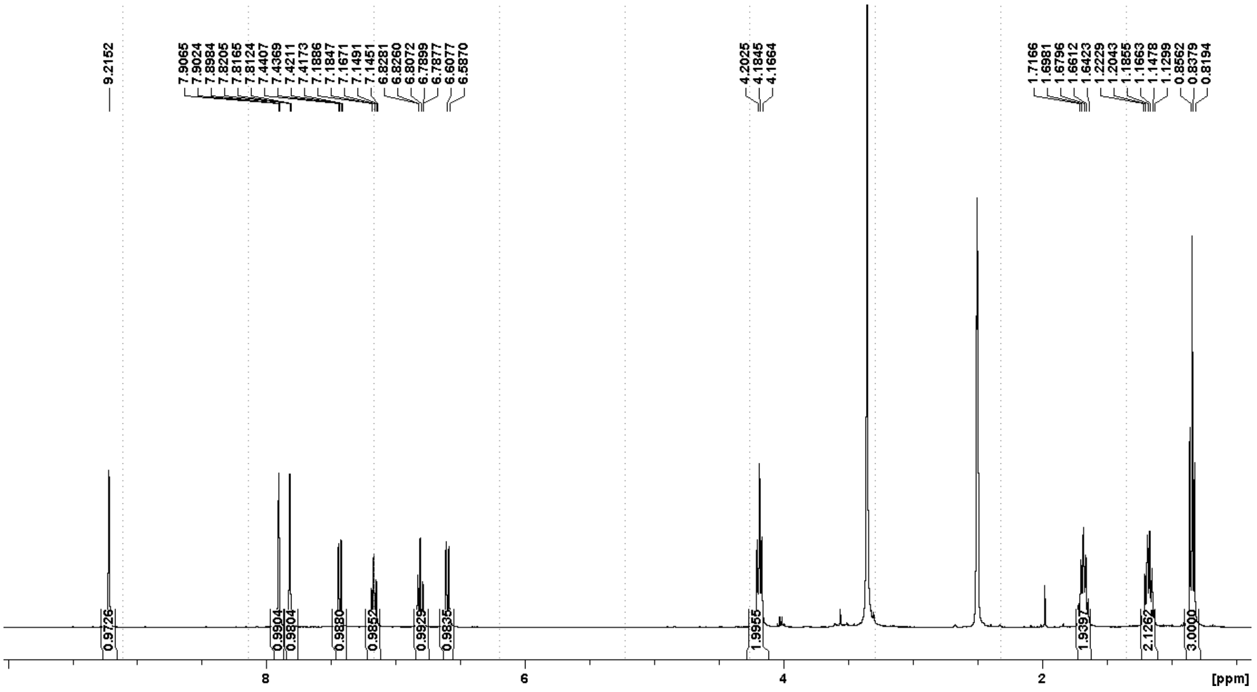

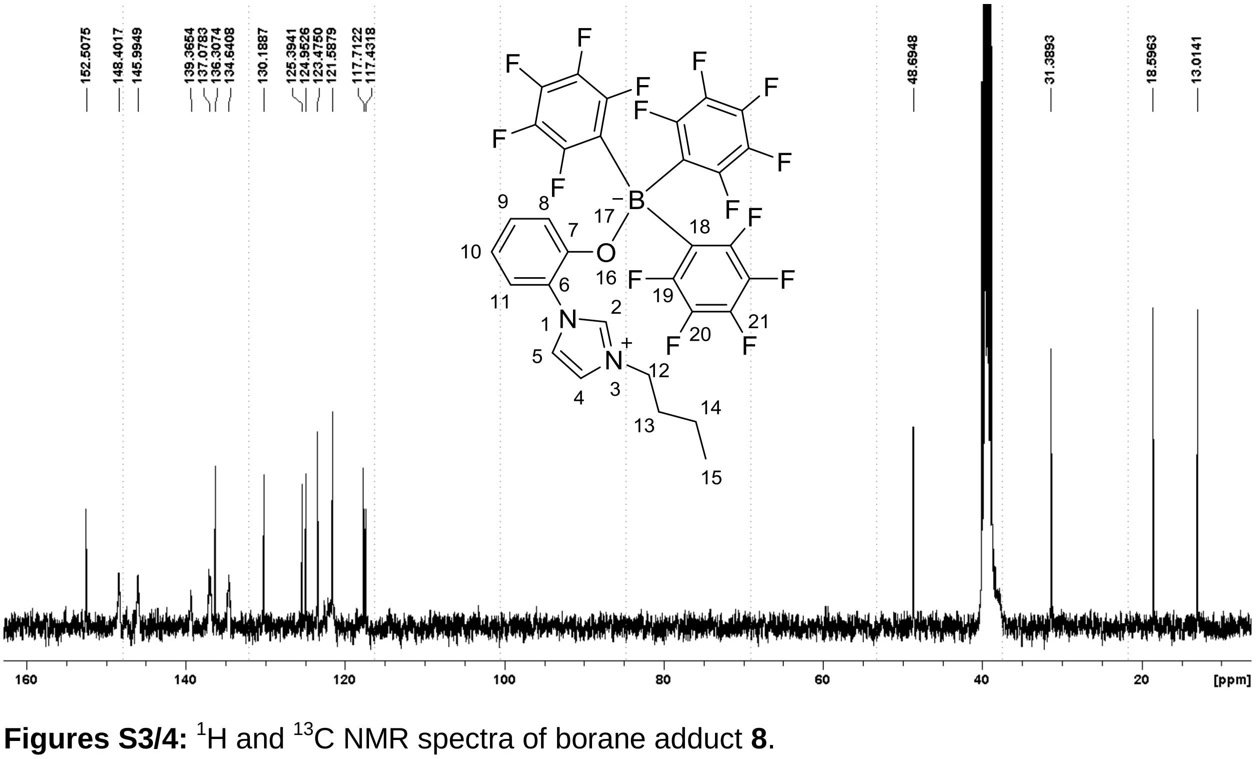

Figures S3/4: ^{1}H and ^{13}C NMR spectra of borane adduct **8**.

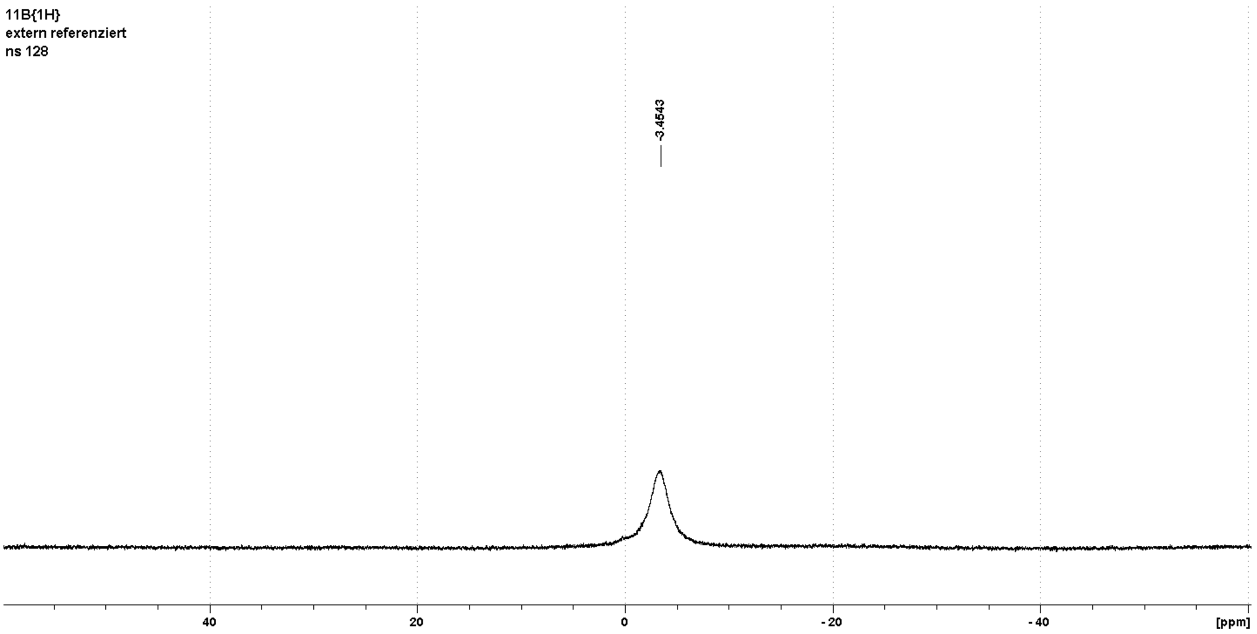

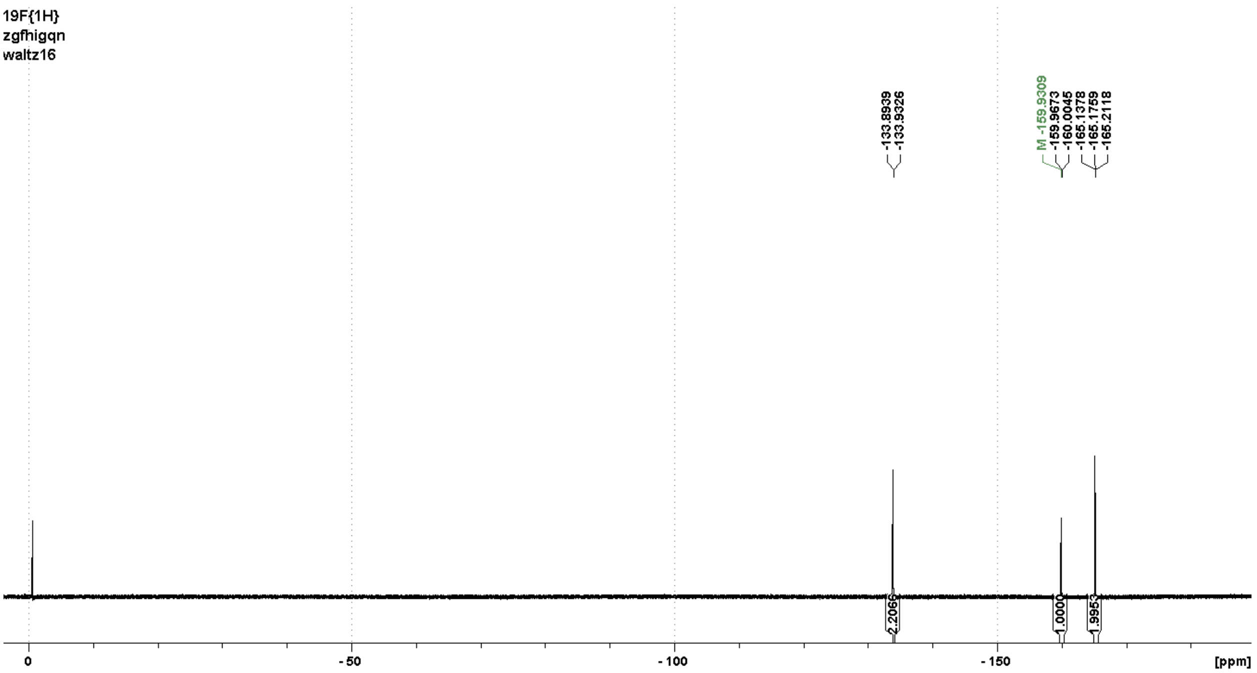

Figures S5/6: ^{11}B and ^{19}F NMR spectra of borane adduct **8**.

Bis(3-butyl-1-(2-hydroxyphenyl)-1*H*-imidazolium-2-yl)gold monochloride (9)

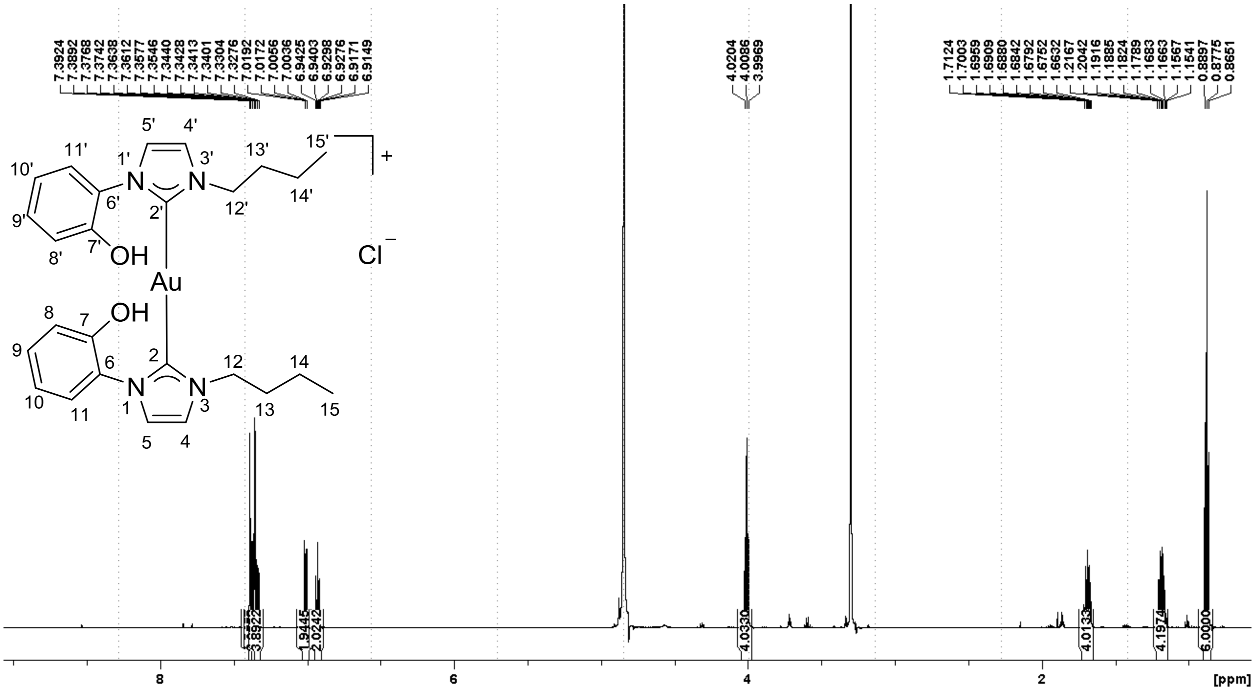

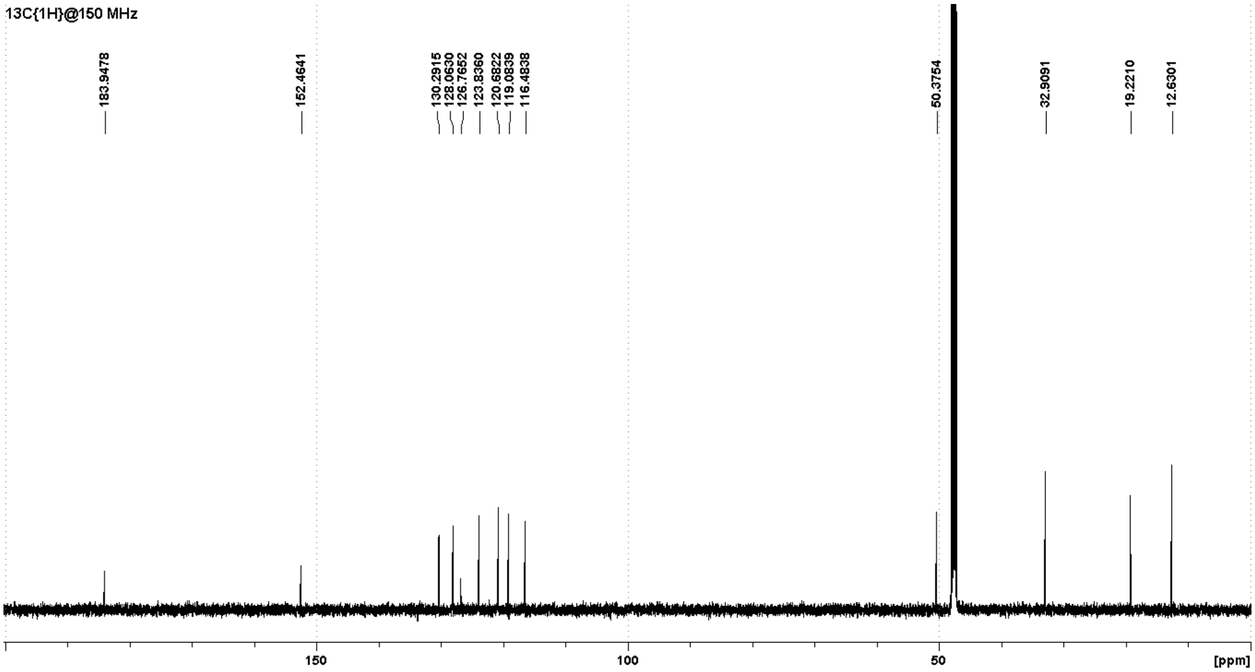

Figures S7/8: ^{1}H and ^{13}C NMR spectra of gold complex **9**.

Potassium bis(3-butyl-1-(2-phenolate)-1*H*-imidazolium-2-yl)gold (10)

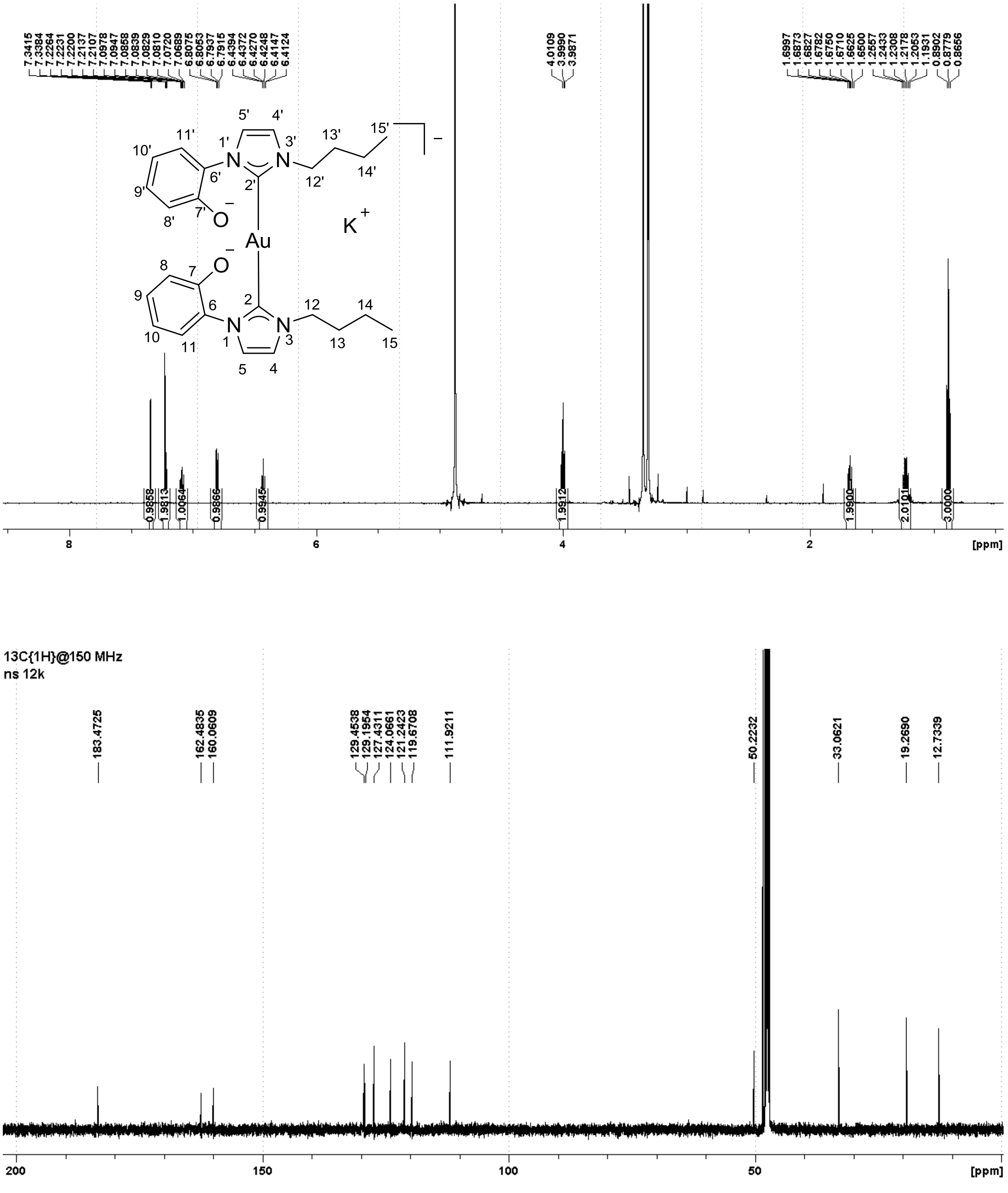

Figures S9/10: ^{1}H and ^{13}C NMR spectra of the anionic gold complex **10**.

Tris(3-butyl-1-(2-oxidophenyl)-1*H*-imidazolium-2-yl)rhodium (11)

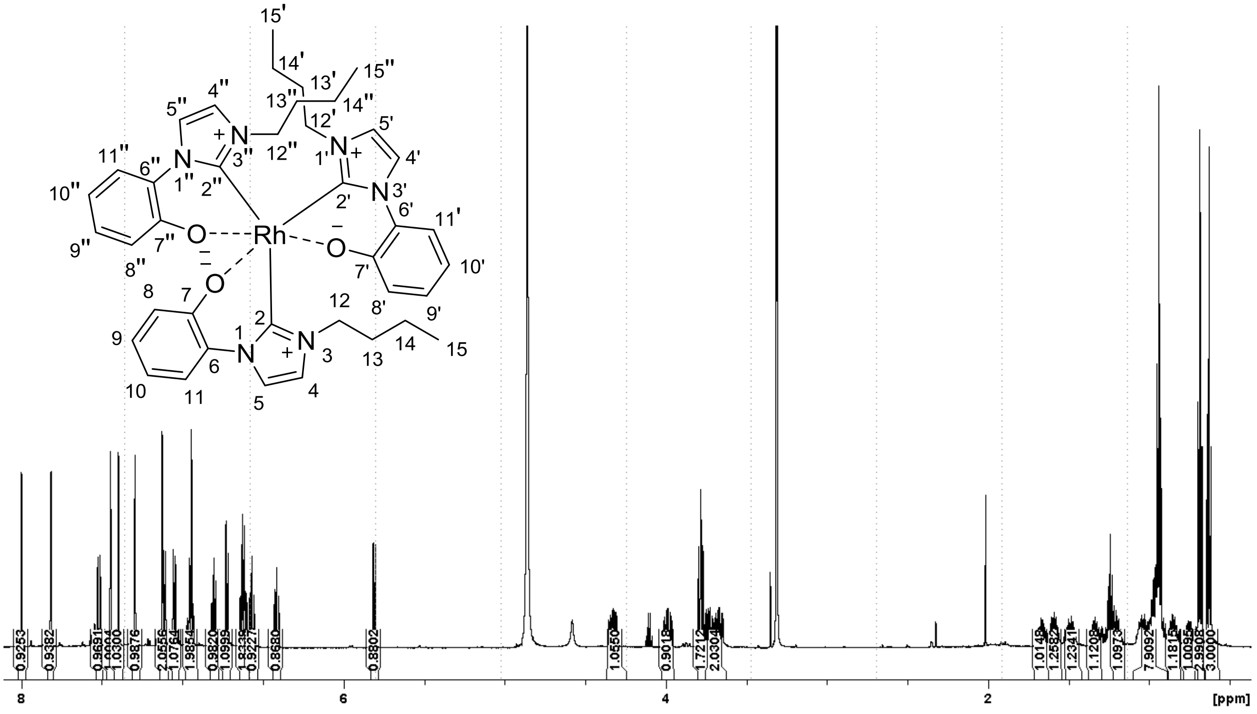

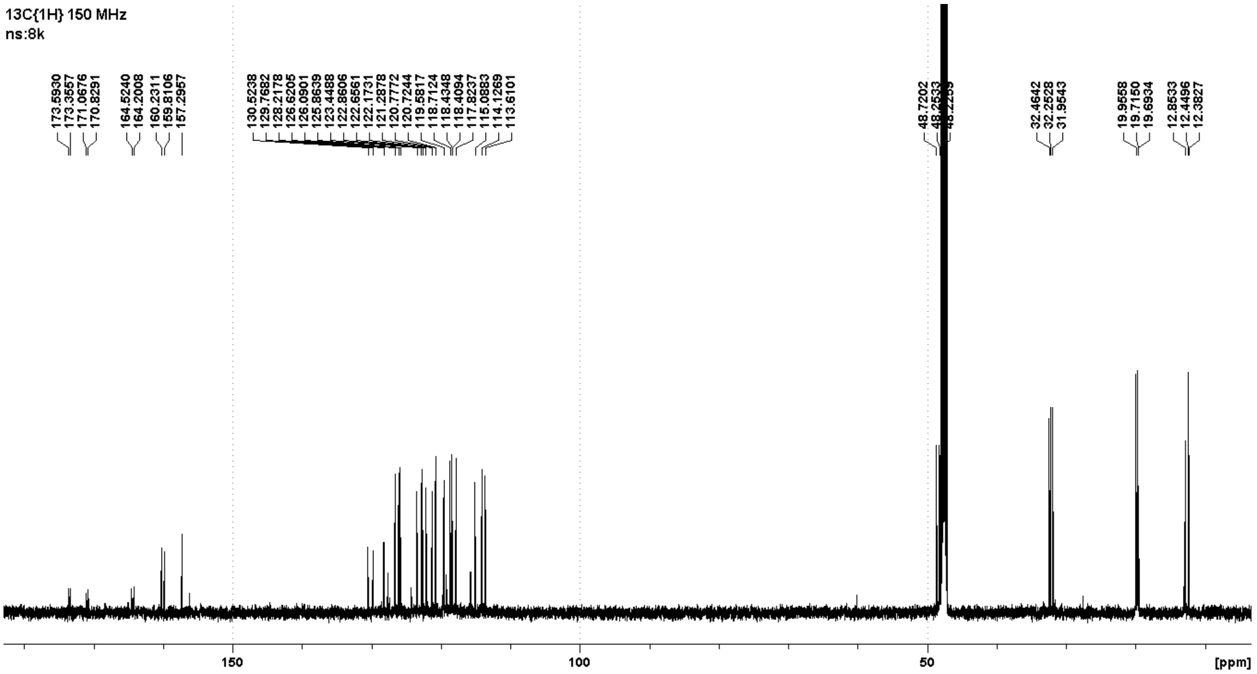

Figures S11/12: ^{1}H and ^{13}C NMR spectra of rhodium complex **11**.

Bis(3-butyl-1-(2-oxidophenyl)-1H-imidazolium-2-yl)nickel (12)

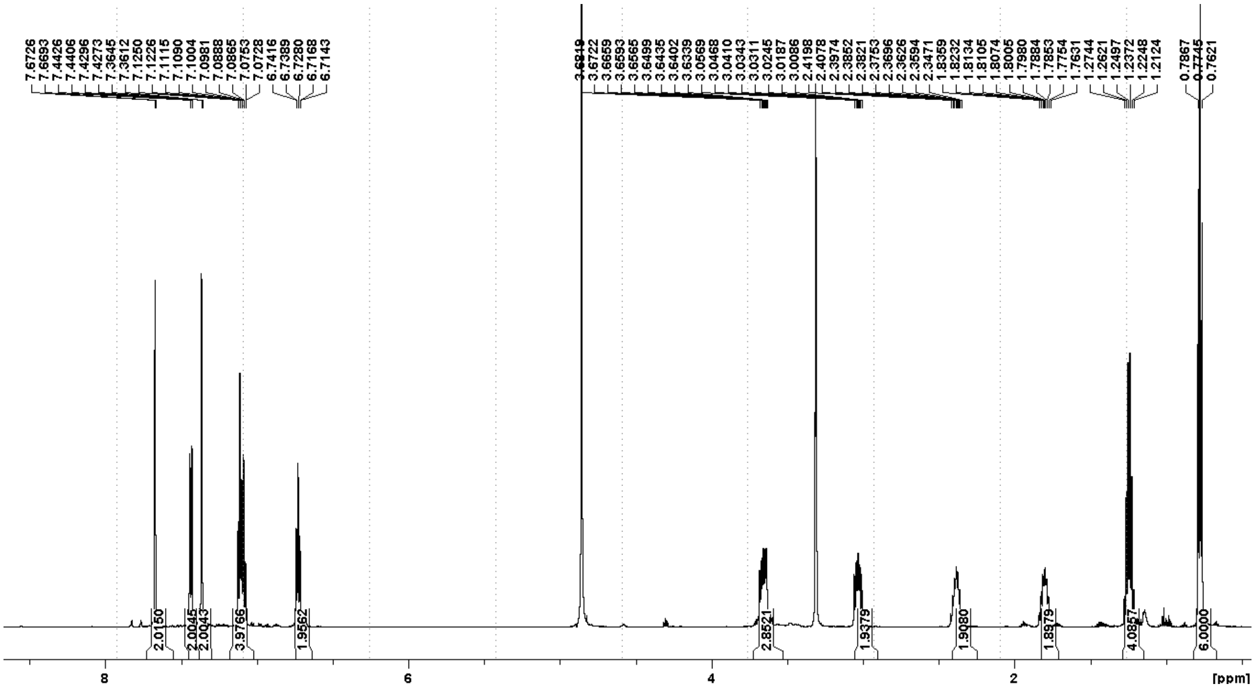

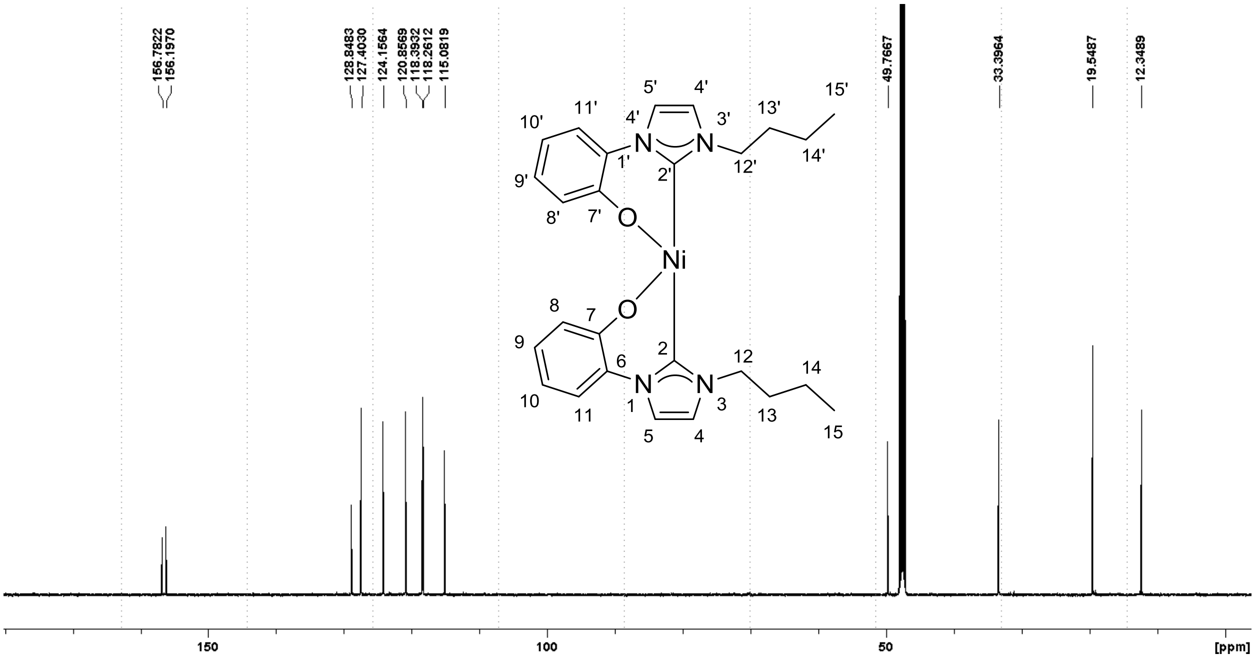

Figures S13/14: ^{1}H and ^{13}C NMR spectra of nickel complex **12**.

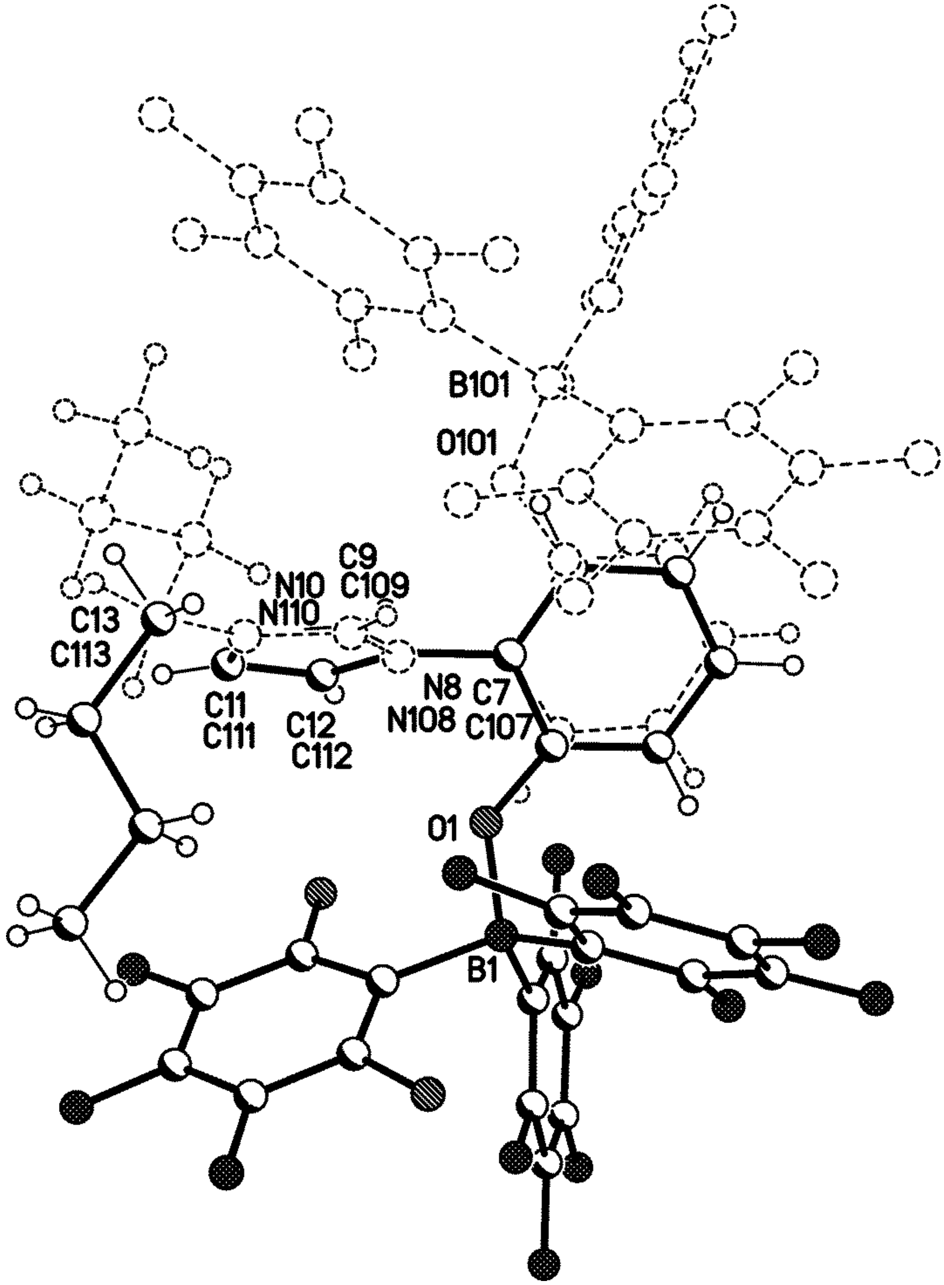

Figure S15: Fit of the two independent molecules of borane adduct **8**

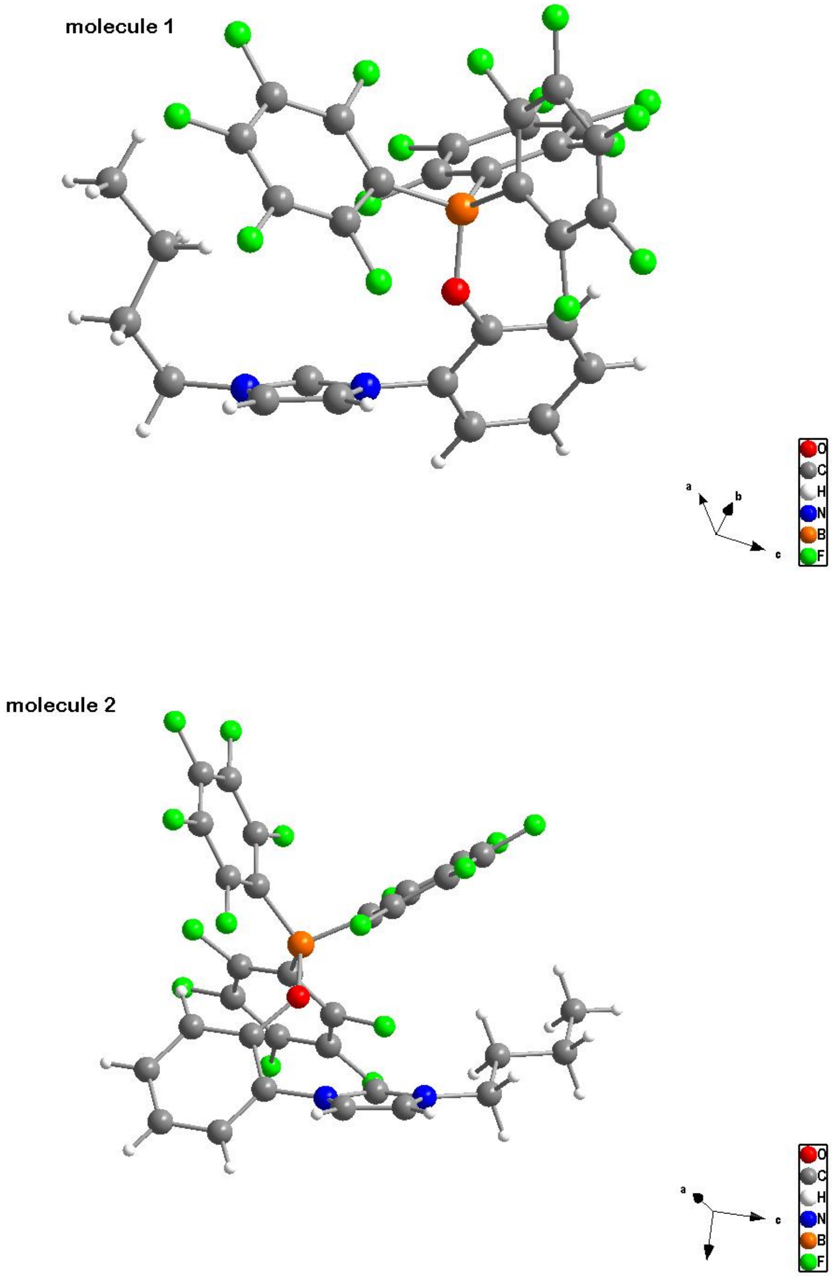

Figure S16: Drawing of the two independent molecules of borane adduct **8**.

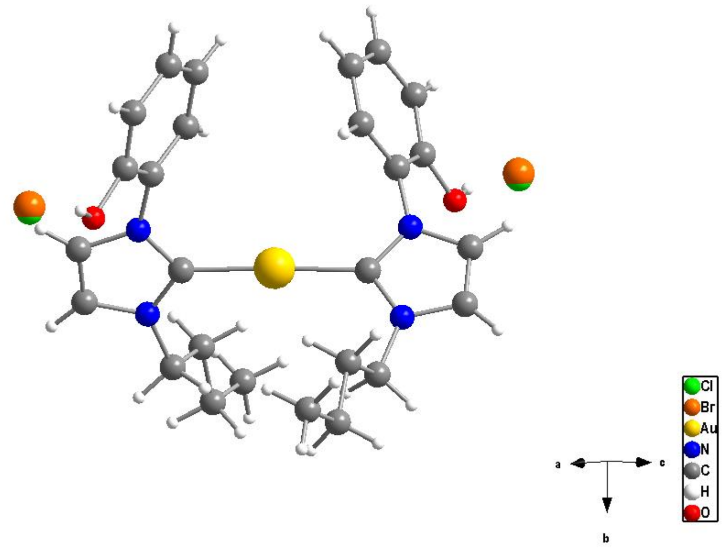

Figure S17: Molecular drawing of gold complex **9**, with disorder of the anion.

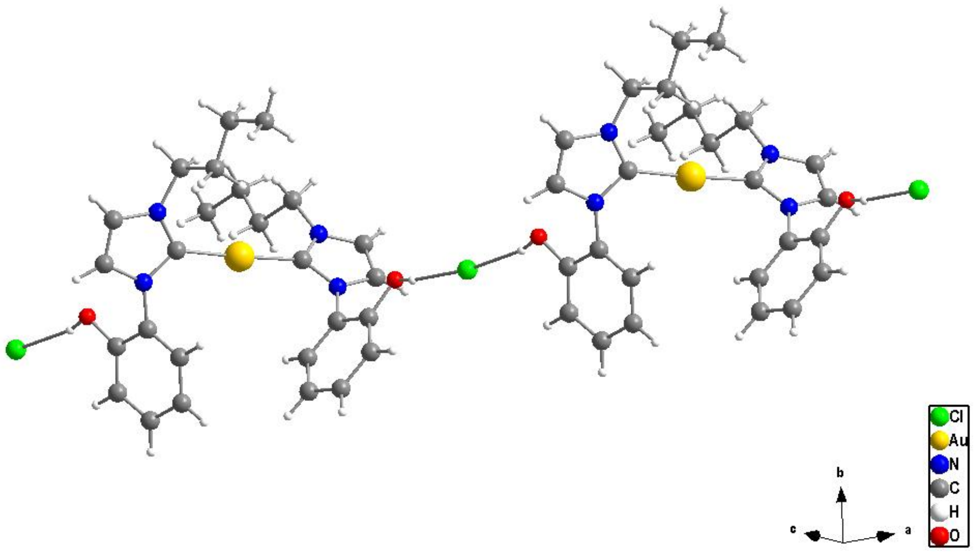

Figure S18: Molecular drawing of the dimer of the coordination polymer of the gold complex **9**; two complexes of **9** are bridged by two hydrogen bonds via the halogene (chloride) counterion.

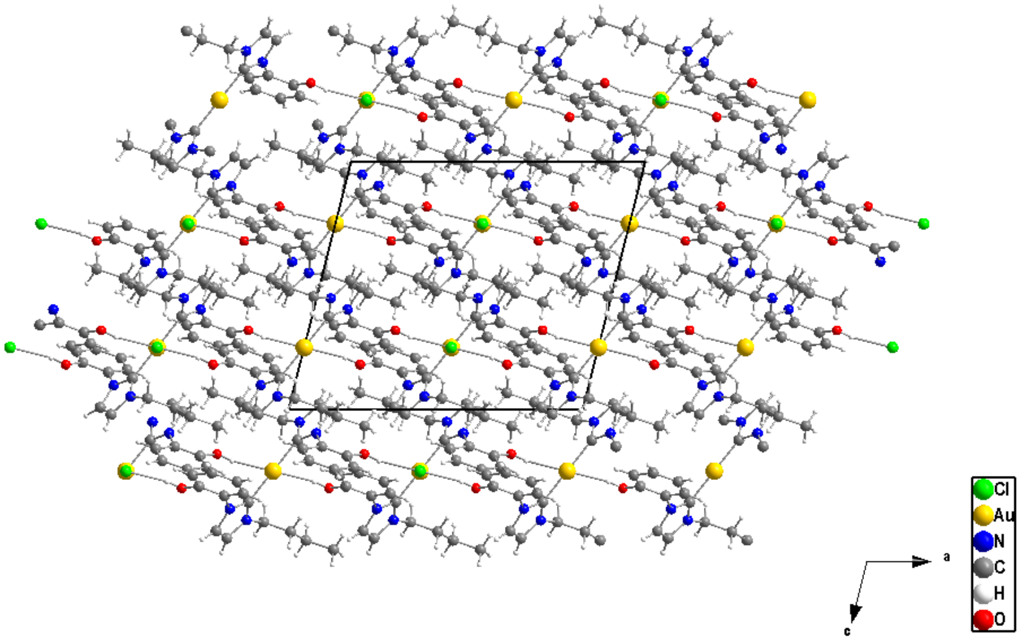

Figure S19: Unit cell of gold complex **9**.

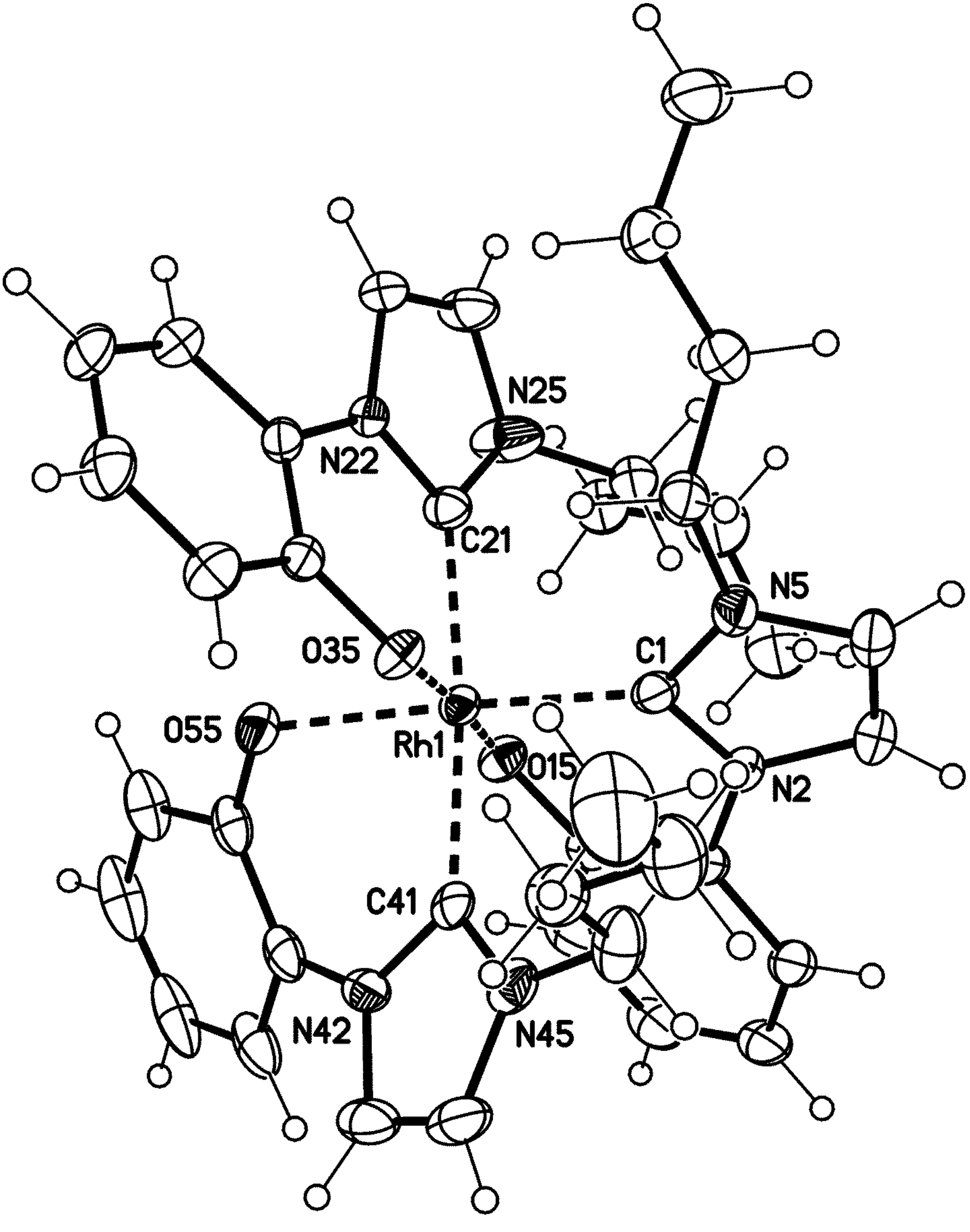

Figure S20: Molecular drawing of the rhodium complex **11** (minor disorder part omitted for clarity, displacement parameters are drawn at 50% probability level).

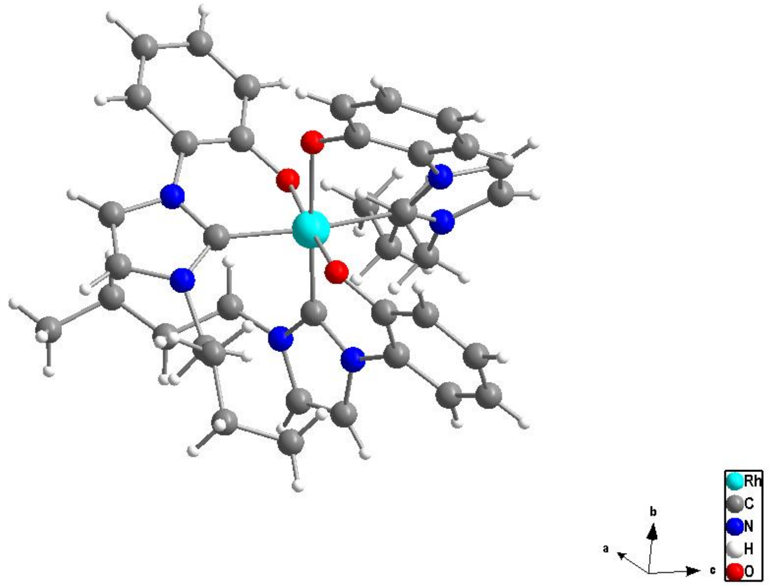

Figure S21: Molecular drawing of the rhodium complex **11**.

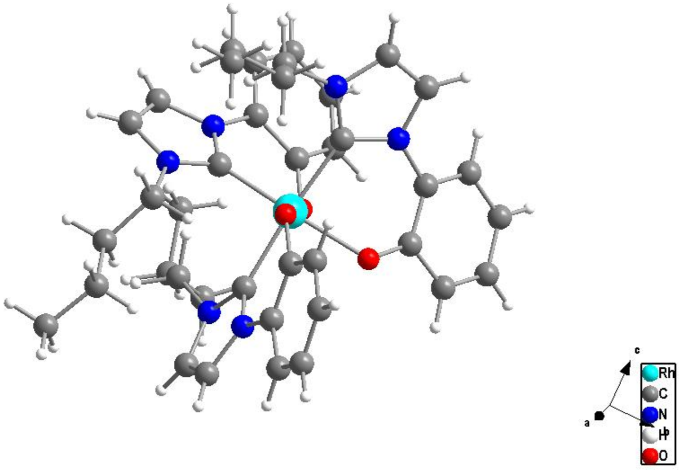

Figure S22: Molecular drawing of the rhodium complex **11**.

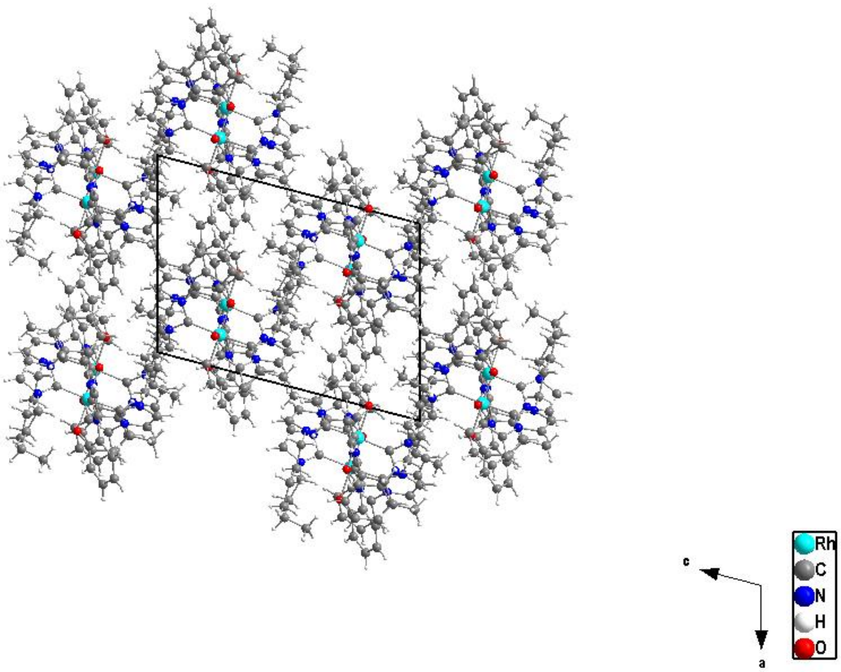

Figure S23: Unit cell of the rhodium complex **11**.

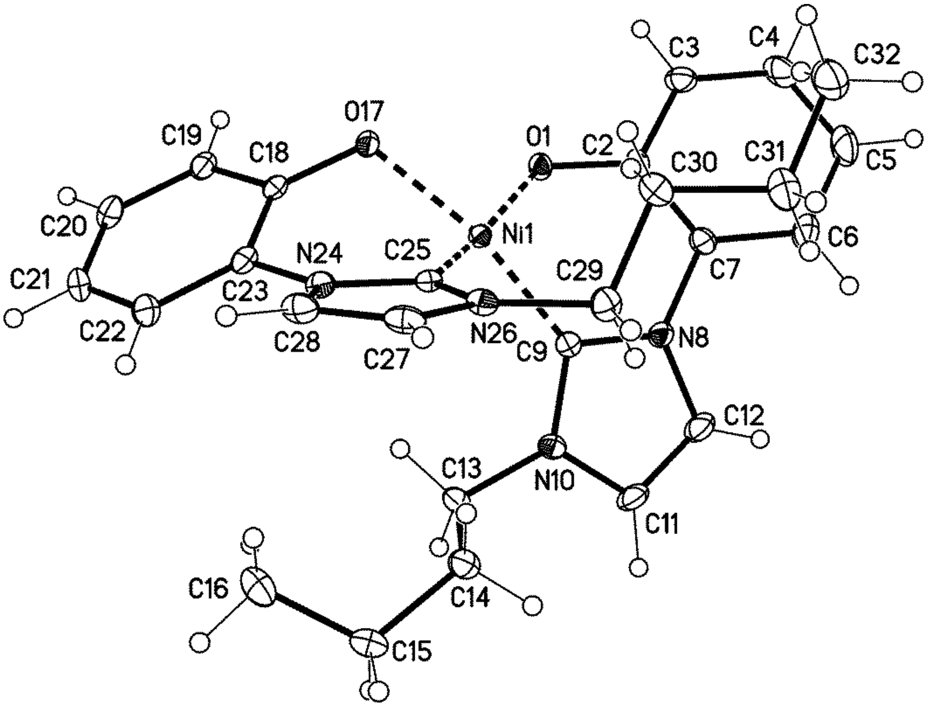

Figure S24: Molecular drawing of one of the crystallographic independent molecules of the nickel complex **12** (minor disorder part omitted for clarity, displacement parameters are drawn at 50% probability level).

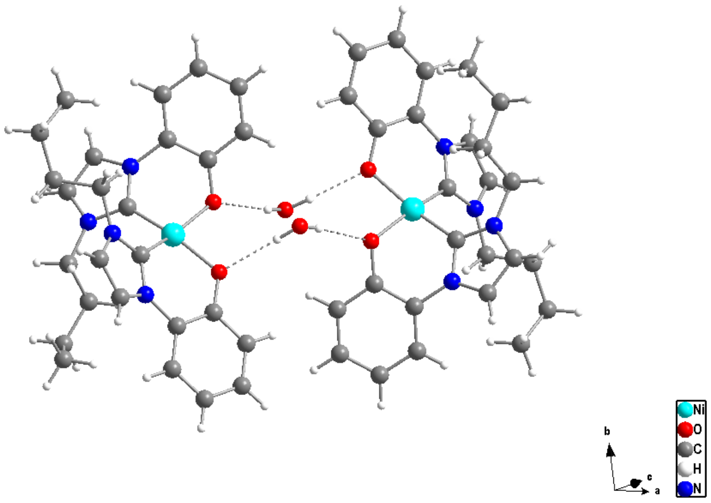

Figure S25: Molecular drawing of the dimeric nickel complex **12**.

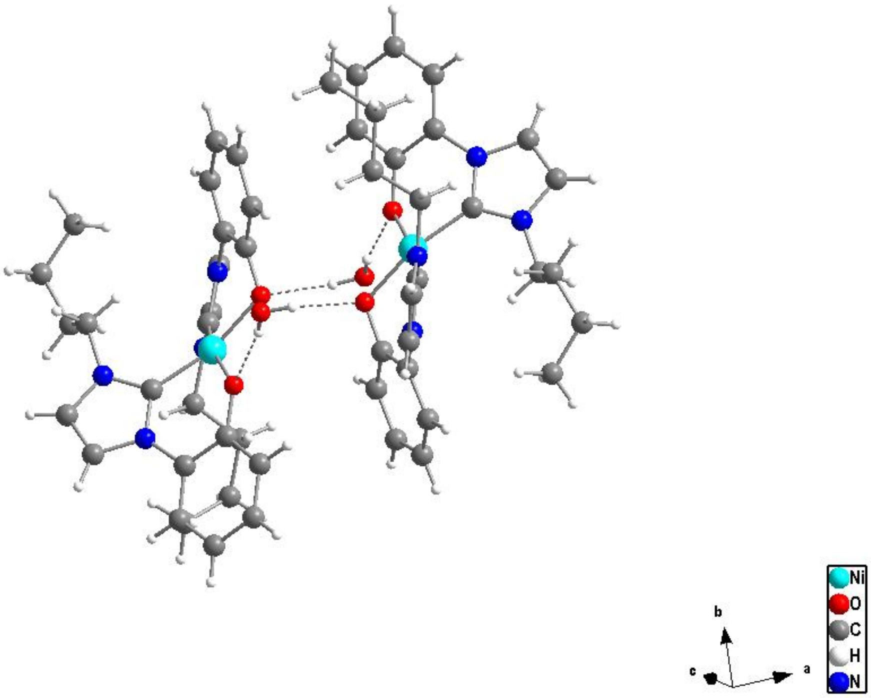

Figure S26: Molecular drawing of nickel complex **12**.
